# Immuno-modulatory effect of probiotic E. coli Nissle 1917 in polarized human colonic cells against Campylobacter jejuni infection

**DOI:** 10.1080/19490976.2020.1857514

**Published:** 2020-12-31

**Authors:** Yosra A. Helmy, Issmat I. Kassem, Gireesh Rajashekara

**Affiliations:** a Food Animal Health Research Program, Department of Veterinary Preventive Medicine, Ohio Agricultural Research and Development Center, the Ohio State University, Wooster, Ohio, USA; b College of Agricultural and Environmental Sciences, Center for Food Safety, University of Georgia, Griffin, Georgia, USA

**Keywords:** Probiotic, *E. coli* Nissle 1917, *Campylobacter*, invasion, intracellular survival, HT-29 cells, innate immune response

## Abstract

*Campylobacter jejuni* is among the leading causes of bacterial foodborne illness. Poultry is the major reservoir and source of human campylobacteriosis. Currently, there is no effective and practical method to decrease *C. jejuni* colonization in chickens or to reduce human infections. Additionally, antibiotic-resistant infections pose a serious public health concern; therefore, antibiotic-alternative approaches are needed to reduce transmission of *C. jejuni* including resistant bacteria from chickens to humans. Here, we evaluated the effect of *E. coli* Nissle 1917 (EcN) on innate responses of polarized HT-29 cells and consequently on *C. jejuni* 81176 infections in HT-29 cells. Pre-treatment of HT-29 cells with EcN for 4 h had a significant effect on the invasion of different *C. jejuni* strains (2 h post-infection) (*P* < .05) and no intracellular *C. jejuni* (24 h post-infection) were recovered. To further understand how EcN mediates its impact on *C. jejuni’s* survival inside the cells, we used Human Antibacterial RT^2^ Profiler^TM^ PCR arrays to profile gene expression in HT-29 cells after treatment with EcN with or without *C. jejuni* 81–176 infection. Our results suggest that pre-treatment of the HT-29 cells with EcN induced the anti-inflammatory cytokines and activated the anti-apoptotic Akt signaling which likely to protect the cells against the proinflammatory and apoptosis responses induced by *C. jejuni*. EcN also positively affected the expression of genes involved in cellular maintenance, growth, development, and proliferation. Further, EcN modulated the expression of genes involved in protective innate immunity, such as TLRs, ERK1/2, p38 MAPK, Ap1, JNK, IL1B, IL17A, and NF-κB signaling.

## Introduction

*Campylobacter jejuni* is one of the most frequent causes of bacterial foodborne gastroenteritis worldwide.^1^ Common symptoms associated with *C. jejuni* infection are diarrhea, fever, and abdominal pain. *C. jejuni* infections can also lead to neurological disorders, such as Guillain-Barre´ and Miller Fisher syndromes.^2^ Chickens are considered the major reservoir and source of human infection.^2^ Currently, there is no effective method available to reduce human infections or to decrease *C. jejuni* colonization in chickens. Moreover, the emergence of antibiotic-resistant *Campylobacter* poses serious public health concerns. Therefore, antibiotic-alternative approaches are needed for sustainable food production.^3^ Recently, the immune-modulatory therapeutics such as probiotic bacteria, as antibiotic alternatives, are used to control various infectious diseases by targeting the host rather than the pathogen to avoid the selection pressure and the evolution of antibiotic-resistant infections.^4^ *Escherichia coli* Nissle 1917 (EcN), a probiotic bacterium, has been shown to have a beneficial effect on human and animal health.^5,6^ EcN persistently colonizes the host and has been shown to; 1) possess immunomodulatory effects,^7,8^ 2) stimulate the intestinal barrier functions,^9^ 3) produce antimicrobial compounds such as bacteriocins and microcins,^10^ and 4) antagonize epithelial colonization and invasion by pathogenic bacteria.^11,12^

Intestinal epithelial cells secrete many mediators such as cytokines, chemokines, and antimicrobial peptides^13^ that are involved in maintaining the host epithelial integrity and protecting the cells from pathogenic bacteria.^14,15^ The innate immune responses are mediated through a pathogen-associated molecular pattern (PAMP) to protect against invading pathogens.^16^ Probiotic bacteria including EcN, have also shown to mediate inflammatory response via the activation of pattern recognition molecules (PRRs), such as toll-like receptors (TLRs) and nucleotide-binding oligomerization domains (NODs), and also through their impact on adaptive response. For example, the TLR pathway stimulates different signaling molecules such as MYD88 and TRAF6, leading to activation of nuclear factor-kB (NF-κB) and extracellular signal-regulated kinase (ERK)/c-Jun-NH2-kinase (JNK)/p38, which induces cytokine secretion, and mediate inflammatory response to control pathogens.^16,17^ Although the immunomodulatory effect of EcN has been demonstrated *in vitro* and *in vivo*,^6,8,18,19^ the impact of EcN on *C. jejuni* invasion to and survival in the intestinal epithelial cells has not been recognized yet. We previously have shown that EcN can reduce *C. jejuni* invasion to and survival in human intestinal cells through the modulation of the cell-to-cell tight junction.^20^ Here, using the antibacterial response RT^2^ PCR array, we investigated the mechanisms by which EcN modulates innate signaling pathways to aid in the control of *C. jejuni* infections. We used polarized human colon cells (HT-29), mimicking *in vivo* intestinal epithelium^21–25^ and has been used previously for the evaluation of *Campylobacter* virulence^26^ and the immune-modulatory effect of the probiotic bacteria.^27^

## Results

### The impact of EcN on adhesion, invasion, and intracellular survival of different C. jejuni isolates in the HT-29 cells

To determine the spectrum of EcN activity, we tested EcN against diverse *C. jejuni* strains that were previously isolated from different hosts (Table 1). Pre-treatment of the HT-29 cells with EcN for 4 h prior to infection with different *C. jejuni* strains resulted in a significant reduction in adhesion, invasion, and intracellular survival of *C. jejuni’s* strains (*P* < .05) in the HT-29 cells. Notably, pretreatment of the cells with EcN resulted in up to 100% clearance with no detectable *C. jejuni* isolated from poultry and starlings (Table 1). Since pretreatment of HT-29 cells with EcN resulted in 93% and 100% reduction in invasion and survival of a well-characterized highly invasive *C. jejuni’s* 81–176 strain,^28^ we used this strain to further evaluate innate responses of HT-29 cells using Human Antibacterial Response RT^2^ Profiler™ PCR Arrays to understand the underlying mechanisms.

**Table 1.** Inhibition of different *C. jejuni’s* strain adhesion to, invasion to and survival in HT-29 cells pretreated with EcN for 4 h

Species		Inhibition %		
	*C.jejuni* strain	Adhesion	Invasion	Intracellular survival
**Human**	81167	40	93.7	100
	NCTC11168	4.8	85.7	88.5
	81116	0	50	100
**Poultry**	Chicken 1	97.5	100	100
	Chicken 2	83.8	94.3	100
	Chicken 3	33.3	100	100
	Chicken 4	54.5	100	100
	Chicken 5	37.5	100	100
**Starlings**	I-Br-14	80	100	100
	F-Br-1	0	100	100
	E-Br-12	9	100	100
**Turkey**	LSE-30	48.9	100	100
	LSW-66	0	16	75
	BOS-114	0	30.8	7.8
**Beef cattle**	Cj-N-33	29	35.7	52
	Cj-M-48	0	0	0
	Cj-M-64	6.7	100	100
**Dairy cattle**	G-D-20	0	70.6	80
	G-D-5	0	71.9	100
	G-D-14	14	28.5	60

* Data for adhesion, invasion, and intracellular survival of *C. jejuni* were presented as percentage of inhibition in EcN-treated cells compared to *C. jejuni* alone infected cells. The experiments were performed two times with four replicates in each experiment.

### EcN enhanced cellular growth and development, proliferation and maintenance, andanti-inflammatory and antimicrobial responses of theHT-29 cells

**Figure 1.**
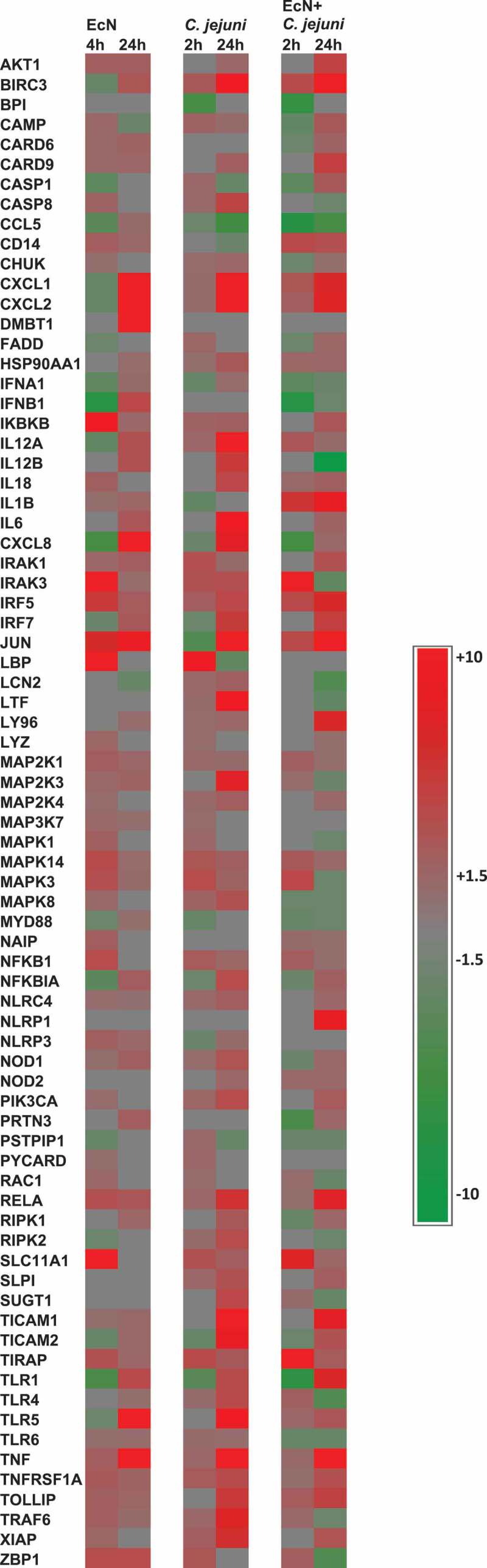
**Heat map showing the transcriptional changes (fold change) in gene expression**. Significant change in gene expression was determined by comparison to untreated cells (no EcN and no *C. jejuni*). A fold change of ± 1.5 ≥ or ≥ 1.5 and a *P* < .05 was used to determine significant differences in gene expression. Red color indicates the increased expression, green color indicates the decreased expression, and gray color indicates expression between ± 1.5 ≥ or ≥ 1.5 folds

**Figure 2.**
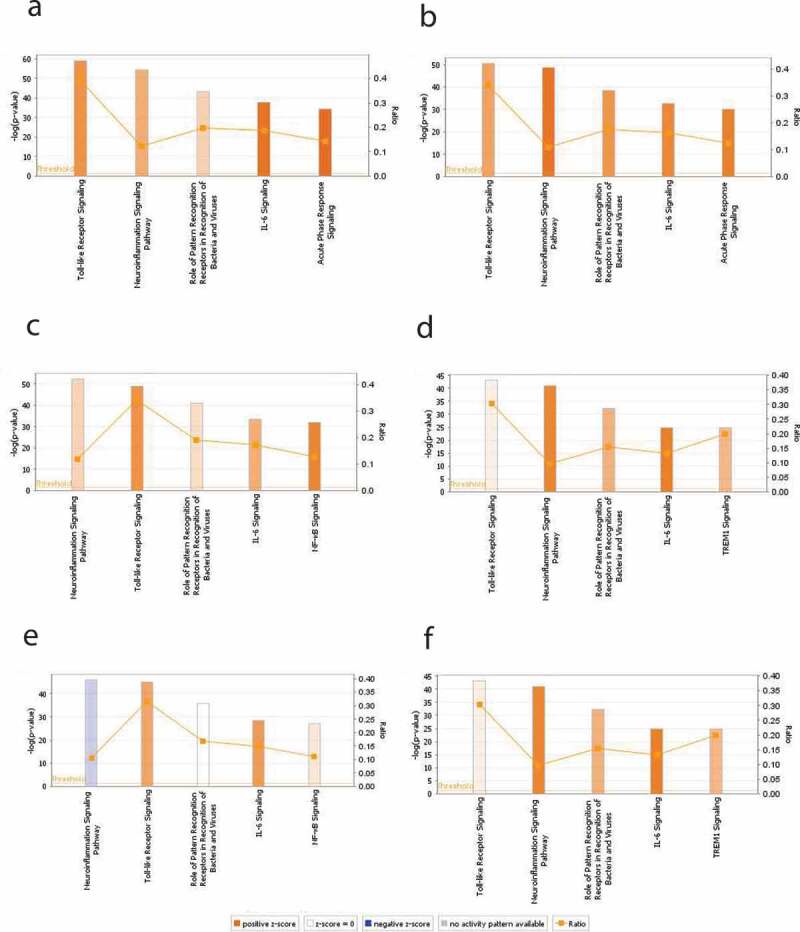
**The top significantly induced canonical pathways (CPs) identified by IPA in HT-29 cells with and without EcN and *C. jejuni***. Expression of genes associated with five significantly modulated CPs at 4 h post-EcN treatment (a), 24 h post-EcN treatment (b), 2 h post *C. jejuni* infection (c), 24 h post *C. jejuni* infection (d), 2 h post-infection of EcN-treated cells with *C. jejuni* (e), and 24 h post-infection of EcN-treated cells with *C. jejuni* (f). The orange and blue colored bars indicate predicted pathway activation, or inhibition, respectively. White bars are CPs with a z-score 0. Gray bars indicate pathways where no prediction can be made. The orange points connected by a thin line represent the Ratio. The ratio is calculated by dividing the number of genes from the data set of differentially expressed gene set that participate in a CP, by the total number of genes in that CP in IPA analysis. The significance values of the CPs are calculated by Fisher’s exact test right-tailed using a -log (*p*-value) cutoff of 1.3, meaning that pathways with a *p*-value ≥ 0.05

Treatment of the HT-29 cells with EcN for 4 h resulted in significant alterations in the expression of 62 genes (44 up- regulated and 18 down-regulated) (*P* < .05). Whereas treatment of the HT-29 cells with EcN for 24 h significantly altered (*P* < .05) the expression of 51 genes (49 up-regulated and 2 down-regulated). Interestingly, most of the down-regulated or slightly induced (*P* < .05) genes at 4 h were up-regulated or highly induced at 24 h after EcN treatment. For example, the expression of CCL5, CXCL1, CXCL2, TLR1, TLR5, TNF, IL8, IL6, IL12A, IL12B, BIRC3, DMBT1, IFNA1, IFNA1, IRF7, JUN, NFKBIA, and PRTN3 were induced (up to 32 folds) at 24 h compared to 4 h post-EcN treatment (Figure 1**; Table S1)**. The highly induced canonical pathways (CP; *P* < .0001) at 4 h and 24 h post-EcN treatment was TLR Signaling, Neuroinflammation Signaling, role of Pattern Recognition Receptors in Recognition of Bacteria and Viruses (PPRs), IL-6, and Acute Phase Response Signaling (Figure 2(a,b)). The top significantly induced CPs at 4 h and 24 h post-treatment, -log (*p*-value), ratio, and their associated genes are listed in **Table S2**.

The prominent cellular and molecular functions (*P* < .01) implicated after 4 h of treatment was Cell-To-Cell Signaling and Interaction, Cell Death and Survival, Cellular Development, Cellular Growth and Proliferation, and Cellular Function and Maintenance. Whereas after 24 h, the most implicated cellular and molecular functions were Cell Death and Survival, Cell-To-Cell Signaling and Interaction, Cellular Function and Maintenance, Cellular Movement, and Cellular Development. The prominent cellular and molecular functions identified at 4 h and 24 h post-EcN treatment, *p*-value overlap, and their associated genes are listed in **Table S3.**

**Figure 3.**
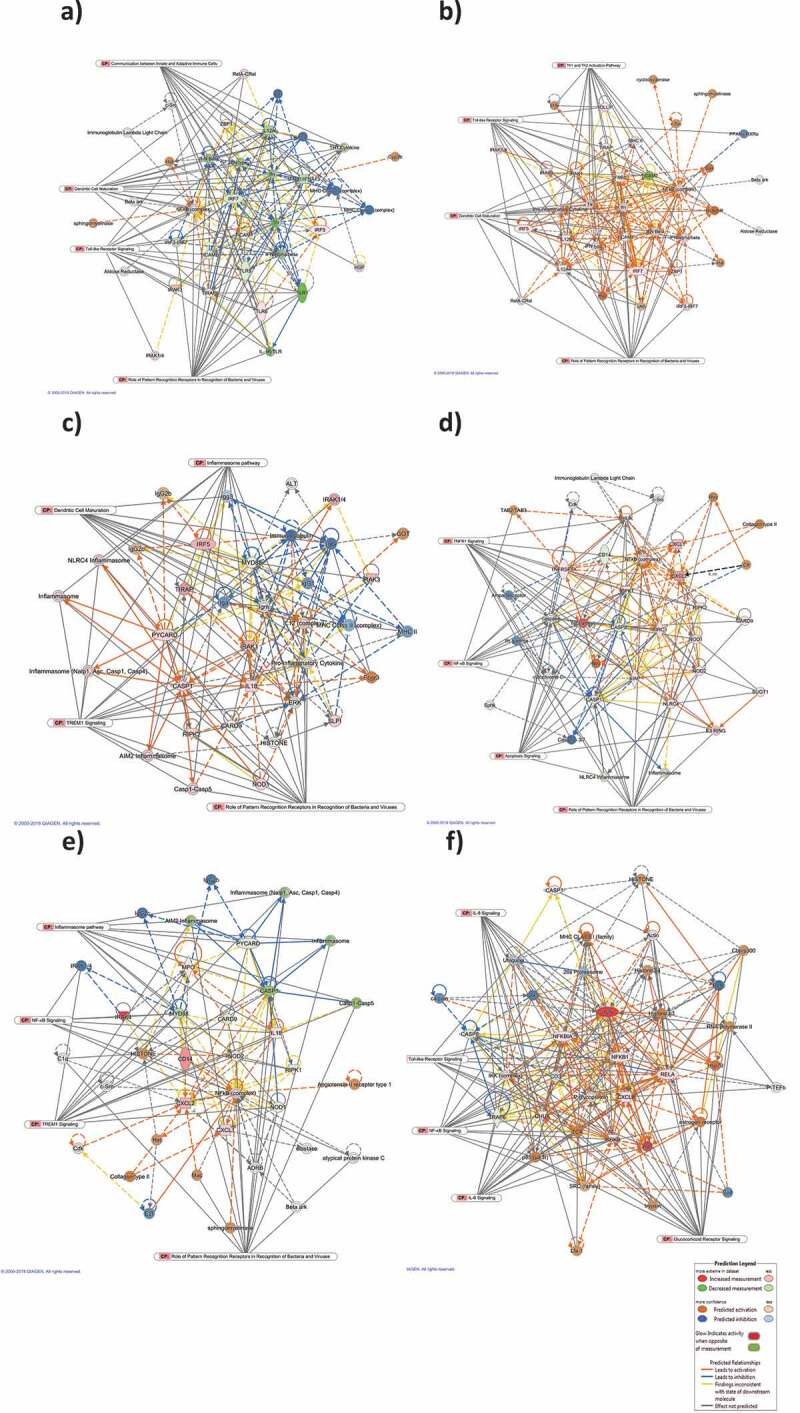
**Top scoring networks with their linked significant canonical pathways**. The interaction maps was derived by plotting interacting genes involved in specific cellular and molecular functions including Protein Synthesis, Cell Morphology, Cellular Function and Maintenance at 4 h post-EcN treatment (a), Cell-To-Cell Signaling and Interaction, Cellular Function and Maintenance, Antimicrobial Response at 24 h post-EcN treatment (b), Cellular Function and Maintenance, Infectious Diseases, Inflammatory Response at 2 h post *C. jejuni* infection (c), Cell Signaling, Cell Death and Survival, Cellular Function and Maintenance at 24 h post *C. jejuni* infection (d), Cell-To-Cell Signaling and Interaction, Cellular Movement, Hematological System Development and Function at 2 h post *C. jejuni* infection of EcN pretreated cells (e), and Cellular Development, Cellular Growth and Proliferation, Connective Tissue Development and Function at 24 h post *C. jejuni* infection of EcN pretreated cells (f). Transcriptional data was projected onto Molecular Activity Predictor interaction map; up-regulated genes are shown in shades of red nodes, down-regulated genes are shown in shades of green nodes and the biological relationship between two nodes is represented as an edge (line). Solid interconnecting lines show the genes that are directly connected and the dotted lines highlight indirect connection between the genes. Relevant canonical pathways (CP) that are highly represented are shown within the box and the relevant genes for each CP were connected to the box by gray line

In addition, gene regulatory networks were built to connect key genes based on the correlation between differentially expressed genes. Networks with at least 10 focus genes were considered to have biological relevance. The top networks modulated after 4 h and 24 h of EcN treatment are listed in Table 2. The top network was connected to top cellular and molecular functions and further analyzed using Molecular Activity Predictor (MAP). The predicted activities of the molecules were linked to the most significant CPs representing the major underlying biological processes. At 4 h post-treatment, the top-scoring network was Protein Synthesis, Cell Morphology, Cellular Function and Maintenance. The main-associated nodes were NFκB complex, IFNA1, TIRAP, IRF7, IFN alfa/beta, TLR1/5, IL12A, TIRAP, TLR, and TICAM1. This network was linked to the CP involved in PPRs (18 genes), TLRS (14 genes), communication between Innate and Adaptive Immune Cells (14 genes), and Dendritic Cell maturation (14 genes). Most of the associated nodes were predicted to be inhibited (Figure 3(a)). Whereas at 24 h, the top-scoring network was Cell-To-Cell Signaling and Interaction, Cellular Function and Maintenance, Antimicrobial Response. The major nodes identified were pro-inflammatory cytokines, NFκB complex, IRF7, TICAM1/2, IL12A/B, IRAK1, TLR, Ifn, IFNB1, IFN beta, IFN type1, and IFN alfa/beta. This network was linked to the CPs involved in PPRs (15 genes), Dendritic Cell maturation (15 genes), TLRS (14 genes), and Th1 and Th2 activation pathway (14 genes). All of the involved nodes were predicted to be activated (Figure 3(b)).

**Table 2.** Top gene regulatory networks associated with the treatment of the HT-29 cells with EcN, *C. jejuni*, and EcN*+ C. jejuni* based on focus molecules and scores*

2 h- post infection			24 h- post infection		
Top network involved	Score	Focus molecules	Top network involved	Score	Focus molecules
**EcN**					
(1) Protein Synthesis, Cell Morphology, Cellular Function and Maintenance	24	12	(1) Cell-To-Cell Signaling and Interaction, Cellular Function and Maintenance, Antimicrobial Response	25	12
(2) Infectious Diseases, Inflammatory Response, Cellular Function and Maintenance	21	11			
***C. jejuni***					
(1) Cellular Function and Maintenance, Infectious Diseases, Inflammatory Response	24	12	(1) Cell Signaling, Cell Death and Survival, Cellular Function and Maintenance	36	16
**EcN*+ C. jejuni***					
(1) Cell-To-Cell Signaling and Interaction, Cellular Movement, Hematological System Development and Function	27	13	(1) Cellular Development, Cellular Growth and Proliferation, Connective Tissue Development and Function	18	10
(2) Cell Death and Survival, Gastrointestinal Disease, Hepatic System Disease	22	11	(2) Cell Death and Survival, Cellular Development, Hematological System Development and Function	18	10

*Only top networks with focus molecules 10 or higher are shown, where “score” reflects number of network eligible molecules; the higher scores indicate that the given network is more likely modulated by different treatment. Focus molecules are the affected genes in different treatments and were considered for generating networks.

The Ingenuity Pathway Analysis (IPA) upstream functional analysis was used to predict the top upstream transcriptional regulators (*P* < .01) from the differentially expressed genes that are either activated or inhibited. The top predicted transcriptional regulators at 4 h post-EcN treatment were IL2, FOXO1, MAPK12, TGFB1. Whereas the top predicted transcriptional regulators at 24 h post-treatment were lipopolysaccharide, TLR4, RELA, NFκB complex, IL2, and P38 MAPK. All of the transcriptional regulators were predicted to be activated. The mechanistic networks associated with the transcriptional regulators at 4 h and 24 h is shown in **Fig. S1A** and **Fig. S1B**, respectively. The top upstream regulators, molecule type, predicted activation state, *P*-value overlap, activation z-score and the target molecules are listed in Table 3.

**Table 3.** Upstream regulator analysis of differentially expressed genes associated with the treatment of the HT-29 cells with EcN, *C. jejuni*, and EcN*+ C. jejuni* based on the overlap of *P* value and z-scores

Upstream Regulator	Molecule type	Predicted activation state	Activation z-score	*P-*value of overlap^*^	Target molecules in dataset
**EcN/4 h post- treatment**					
IL2	Cytokine	Activated	2.467	5.49E-09	BIRC3,CARD9,CASP1,CCL5,CXCL8,IL18,IL1B,JUN,MAP2K1,NFKB1,TNF,TNFRSF1A,TRAF6
FOXO1	Transcription regulator	Activated	2.353	3.85E-07	BIRC3,CASP8,CXCL8,IL18,IL1B,IRF5,JUN,RELA,TNF,TRAF6
MAPK12	Kinase	Activated	2.204	3.33E-09	IFNB1,IL12A,IL1B,JUN,TNF
TGFB1	Growth factor	Activated	2.059	4.23E-12	AKT1,CAMP,CASP1,CASP8,CCL5,CD14,CXCL1,CXCL8,HSP90AA1,IL12A,IL18,IL1B,IRAK3,JUN,MAP2K1,MAP2K3,MAPK1,MAPK14,MAPK3,MYD88,NFKBIA,NLRP3,RAC1,TNF,XIAP
dextran sulfate	Chemical drug	Activated	2.055	2.81E-08	CCL5,CD14,CXCL8,IL12A,IL18,IL1B,IRF7,LY96,TNF
**EcN/24 h post- treatment**					
lipopolysaccharide	Chemical drug	Activated	3.703	8.97E-31	BIRC3,CARD6,CCL5,CD14,CXCL1,CXCL2,CXCL8,DMBT1,IFNA1/IFNA13,IFNB1,IL12A,IL12B,IL1B,IL6,IRAK1,IRAK3,IRF5,IRF7,JUN,LCN2,LY96,LYZ,MAP3K7,MAPK8,NFKBIA,NLRP3,NOD1,PRTN3,PYCARD,RELA,TICAM1,TIRAP,TLR1,TLR5,TLR6,TNF,TNFRSF1A,TOLLIP,TRAF6
TLR4	Transmembrane receptor	Activated	3.624	2. 79E-17	CCL5,CD14,CXCL2,CXCL8,IFNA1/IFNA13,IFNB1,IL12A,IL12B,IL1B,IL6,IRAK3,IRF7,LCN2,NFKBIA,RELA,TNF,TRAF6
RELA	Transcription regulator	Activated	3.365	4.03E-15	BIRC3,CCL5,CD14,CXCL1,CXCL2,CXCL8,IFNB1,IL12A,IL12B,IL1B,IL6,IRF7,JUN,NFKBIA,RELA,TNF
NFkB (complex)	Complex	Activated	3.179	2.62E-17	BIRC3,CCL5,CXCL1,CXCL2,CXCL8,DMBT1,IFNA1/IFNA13,IFNB1,IL12A,IL12B,IL1B,IL6,IRF7,JUN,LCN2,NFKBIA,RELA,TNF,TNFRSF1A,TOLLIP
IL2	Cytokine	Activated	3.177	1.13E-09	BIRC3,CARD6,CARD9,CCL5,CXCL8,IL12B,IL1B,IL6,JUN,MAP2K1,TNF,TNFRSF1A,TRAF6
P38 MAPK	Group	Activated	3.036	6.23E-12	CCL5,CXCL1,CXCL2,CXCL8,IFNB1,IL12A,IL12B,IL1B,IL6,IRF7,JUN,TNF,TNFRSF1A
***C. jejuni/*2 h post- infection**					
wortmannin	Kinase inhibitor	Activated	2.534	1.93E-10	AKT1,CCL5,CXCL8,IFNB1,IL12A,IL1B,JUN,NFKB1,NFKBIA,TNF
CSF3	Cytokine	Activated	2.475	5.38E-17	BIRC3,CCL5,CXCL2,CXCL8,IL12A,JUN,LBP,LCN2,LTF,LY96,LYZ,TLR4,TNF,TNFRSF1A
LEP	Growth factor	Inhibited	−2.012	3.60E-05	CCL5,FADD,IL12A,IL1B,JUN,RAC1,TNF,TNFRSF1A
Jnk	Group	Inhibited	−2.136	1.56E-07	BIRC3,CCL5,CXCL8,IFNB1,IL1B,JUN,TNF,XIAP
IL5	Cytokine	Inhibited	−2.236	1.30E-03	CXCL8,IFNB1,IRF7,TLR1,TNF
***C. jejuni/*24 h post- infection**					
IL4	Cytokine	Activated	3.114	1.69E-12	CCL5,CD14,CXCL1,CXCL2,CXCL8,IL12A,IL12B,IL1B,IRF5,IRF7,JUN,MAP2K1,NFKB1,NFKBIA,PSTPIP1,TICAM1,TLR4,TNF
EZH2	Transcription regulator	Activated	2.525	1.46E-06	BIRC3,CCL5,CXCL1,CXCL2,CXCL8,LCN2,NFKBIA,TNF
HGF	Growth factor	Activated	2.435	7.66E-13	AKT1,BIRC3,CASP1,CASP8,CCL5,CXCL2,CXCL8,IL12B,IL1B,LCN2,MAP2K3,MAPK14,NFKB1,TNF,TNFRSF1A
IGF1	Growth factor	Activated	2.394	6.88E-12	AKT1,BIRC3,CAMP,CCL5,CXCL8,IL1B,JUN,LCN2,NFKBIA,RELA,SLPI,TLR4,TNF,XIAP
IL1A	Cytokine	Activated	2.386	6.99E-18	BIRC3,CCL5,CXCL1,CXCL2,CXCL8,IL12B,IL1B,IRAK1,JUN,LCN2,NFKB1,NFKBIA,RELA,TICAM1,TNF
**EcN*+ C. jejuni/*2 h post- infection**					
dextran sulfate	Chemical drug	Activated	3.092	3.14E-11	CCL5,CD14,CXCL2,CXCL8,IL12A,IL18,IL1B,IRF7,MPO,TLR4,TNF
EIF2AK3	Kinase	Activated	2.2	9.06E-06	BIRC3,IL1B,JUN,TNF,XIAP
FCGR2A	Transmembrane receptor	Activated	2.081	1.03E-08	CCL5,CXCL2,CXCL8,IL1B,TNF
LEP	Growth factor	Activated	2.012	2.11E-06	CCL5,CD14,FADD,IL12A,IL1B,JUN,RAC1,TNF,TNFRSF1A
TLR4	Cytokine	Activated	0.828	1.77E-24	BPI,CCL5,CD14,CXCL2,CXCL8,IFNA1/IFNA13,IFNB1,IL12A,IL18,IL1B,IRAK3,IRF7,MYD88,NFKB1,NFKBIA,NOD2,RAC1,RELA,TLR4,TNF,TRAF6,XIAP
**EcN*+ C. jejuni/*24 h post- infection**					
RELA	Transcription regulator	Activated	3.198	1.3E-19	BIRC3,CAMP,CASP8,CCL5,CD14,CXCL1,CXCL2,CXCL8,IFNB1,IL12A,IL12B,IL1B,IL6,IRF7,JUN,NFKB1,NFKBIA,NOD2,RELA,TNF,XIAP
NFkB (complex)	Complex	Activated	2.958	6.62E-21	BIRC3,CAMP,CASP8,CCL5,CXCL1,CXCL2,CXCL8,IFNA1/IFNA13,IFNB1,IL12A,IL12B,IL1B,IL6,IRF7,JUN,LCN2,NFKB1,NFKBIA,RELA,SLPI,TLR4,TNF,TNFRSF1A,TOLLIP,XIAP
MYD88	Other	Activated	2.688	3.19E-19	CCL5,CD14,CXCL1,CXCL2,CXCL8,IFNA1/IFNA13,IFNB1,IL12A,IL12B,IL1B,IL6,IRF7,JUN,NFKB1,NFKBIA,NOD2,RELA,TNF
lipopolysaccharide	Chemical drug	Activated	2.565	1.96E-37	BIRC3,CAMP,CARD6,CASP1,CASP8,CCL5,CD14,CHUK,CXCL1,CXCL2,CXCL8,IFNA1/IFNA13,IFNB1,IL12A,IL12B,IL1B,IL6,IRAK1,IRAK3,IRF5,IRF7,JUN,LBP,LCN2,LTF,LY96,LYZ,MAPK8,MPO,MYD88,NFKB1,NFKBIA,NOD1,NOD2,PRTN3,RELA,RIPK2,SLC11A1,SLPI,TICAM1,TIRAP,TLR1,TLR4,TLR5,TLR6,TNF,TNFRSF1A,TOLLIP,TRAF6
IL1A	Cytokine	Activated	2.542	2.17E-17	BIRC3,CCL5,CXCL1,CXCL2,CXCL8,IL12B,IL1B,IL6,IRAK1,JUN,LCN2,NFKB1,NFKBIA,RELA,TICAM1,TNF

*An overlap *p*-value is computed based on significant overlap between genes in the dataset and known targets regulated by the transcriptional regulator. The degree of the z-score is the degree to which a factor is activated or inhibited.

Our analysis highlights the importance of EcN in modulating the protective anti-inflammatory response of the HT-29 cells through the activation of various signaling pathways including NFκB, ERK1/2, p38MAPK, Ap1, JNK, TLR4, IL1B, and IL17A. The activation of downstream signaling molecules such as p38 MAPK, JNK, and NF-κB lead to the activation of transcription regulators like c-Fos and c-Jun, and consequently induction of several proinflammatory cytokines, leading to antibacterial and antiviral responses. Further, EcN induced TLRs signaling which mediates infection control, and dendritic cells (DC) maturation, an important initiator of immune responses to microbial pathogens.

### C. jejuni induces cellular proinflammatory response and apoptosis, and disrupts the epithelial barrier integrity

Infection of the HT-29 cells with *C. jejuni* for 2 h (invasion) affected the expression of 58 genes (46 up-regulated and 12 down-regulated). Whereas infection of the cells with *C. jejuni* for 24 h (intracellular survival) affected the expression of 60 genes (55 up-regulated and 5 down-regulated) (*P* < .05). Notably, 35 down-regulated or slightly induced genes at 2 h were up-regulated or highly induced (up to 43 folds) at 24 h post-infection (Figure 1**; Table S1**). In addition, the highly induced CPs (*P* < .0001) at 2 h post-infection were Neuroinflammation Signaling, TLRs, PPRs, IL-6, and NF-kB Signaling (Figure 2(c)); while at 24 h, the highly induced CPs (*P* < .0001) were TLRs, Neuroinflammation Signaling, PPRs, IL6, and TREM1 Signaling (Figure 2(d)). The top-induced CPs at 2 h and 24 h post-infection, *p*-value, ratio, and their associated genes are listed in **Table S2**. The PPRs, IL6, and TREM1 CPs were highly induced at 24 h compared to 2 h post-infection. PPRs such as TLRs use an adapter protein (TRAF6 and MYD88) to activate NF-κB that induces the proinflammatory cytokines in response to *C. jejuni* infection via the process of phagocytosis and apoptosis or directly induces the production of proinflammatory cytokines by activating NF-κB.

The prominent cellular and molecular functions (*P* < .01) induced 2 h after *C. jejuni* infection was cell Death and Survival, Cell-To-Cell Signaling and Interaction, Cellular Development, Cellular Growth and Proliferation, and Cellular Function and Maintenance. Whereas after 24 h of infection, the most implicated functions were Cell Death and Survival, Cell-To-Cell Signaling and Interaction, Cellular Development, Cellular Function, and Maintenance, and Cell Signaling. All the induced functions at 2 h and 24 h, post-infection, *p*-value overlap, and their associated genes are listed in **Table S3**.

Additionally, at 2 h post-infection, the top regulatory networks were Cellular Function and Maintenance, Infectious Diseases, Inflammatory Response. The major nodes connecting the genes were MYD88, a pro-inflammatory cytokine, CARD9, ERK, CASP1, IL18, IL12, PYCARD, IRAK1, and the inflammasome. Most of the nodes were predicted to be activated. This network was linked to TREM1 (13 genes), PPRs (13 genes), Dendritic Cell Maturation (12 genes), and inflammasome (10 genes) CPs. The inflammasome regulates the activation of caspase-1 to induce inflammation in response to infectious microbes (Figure 3(c)). Activation of caspase-1 will result in the activation of proinflammatory cytokines (IL-1) that induce IL-1β and IL-18 and cause inflammatory cell death. At 24 h post-infection, the top-scoring network was Cell Signaling, Cell Death and Survival, Cellular Function and Maintenance. The major-identified nodes were NFκB complex, CXCL1/2, CHUK, NOD1/2 TNFRSFIA, NLRC4, TNF, CASP1/8, and CD14, which are predicted to be activated. This network was linked to the CPs involved in PPRs (13 genes), apoptosis (13 genes), TNFR1 (13 genes), and NF-κB signaling (9 genes) (Figure 3(d)). The top regulatory networks, score, and the associated genes are listed in Table 2.

The top predicted transcriptional regulators (*P* < .01) at 2 h post-infection were wortmannin, CSF3 which is predicted to be activated, LEP, JNK, and, IL5 which are predicted to be inhibited. These regulators activate the NFκB complex. Whereas at 24 h transcription regulators such as IL4, EZH2, HGF and IGF1, and IL1A were predicted to be activated. The mechanistic networks associated with the transcriptional regulators at 2 h and 24 h are shown in Fig. S1C and Fig. S1D, respectively. The top upstream regulators, molecule type, activation state, *p*-value overlap, z-score, and the target molecules are shown in Table 3.

Overall, our analysis showed that *C. jejuni* activated NF-κB at early and late stages of infection, leading to induction of proinflammatory cytokines (TNFα and IL-8), which negatively affect the epithelial barrier integrity^29^ and consequently induce cellular apoptosis. Additionally, *C. jejuni* inhibited p38MAPK, ERK1/2, and JNK which regulate cellular tight junction and epithelial barrier function.^30^

### EcN induced protective anti-inflammatory response that modulates proinflammatory immune response caused by C. jejuni infection on HT-29 cells

Infection of the EcN pretreated HT-29 cells with *C. jejuni* for 2 h (invasion) resulted in a significant alteration in the expression of 51 genes (31 up-regulated and 20 down-regulated) (*P* < .05). While at 24 h post-infection (intracellular survival), the expression of 70 genes (52 up-regulated and 18 down-regulated) was altered (*P* < .05). Notably, 30 genes were induced (up to 22 folds) at 24 h compared to 2 h post-infection (Figure 1**; Table S1**). Interestingly, treatment of the HT-29 cells with EcN before infection with *C. jejuni* reduced the expression of CASP8, IFNA1, IFNB1, IL12B, IL18, IL6, IL8, IRAK3, JUN, LCN2, LTF, MAP2K3, MAPK1 (ERK), MAPK3, MAPK8, MAPK14 (P38), NFKBIA, NOD1, RIPK2, TICAM1, TICAM2, TLR4, TLR5, TNF, TRAF6, and XIAP encoding genes (up to 30 folds) compared to *C. jejuni* alone (Figure 1**; Table S1; *P* < .05**). The majority of these genes regulate proinflammatory response after *C. jejuni’s* infection.

The highly induced CPs (*P* < .0001) at 2 h post-infection were Neuroinflammation Signaling, TLRs, PPRs, IL6, and NF-kB Signaling (Figure 2(e)); while at 24 h, the highly induced CPs (*P* < .0001) were TLRS, Neuroinflammation Signaling, PPRs, IL6, and TREM1 Signaling (figure 2(f)). TLRs, PPRs, IL6, TREM1, and NF-κB CPs were highly activated at 24 h compared to 2 h post-infection. The top-induced CPs at 2 h and 24 h post-infection, *p*-value, ratio, and their associated genes are listed in **Table S2.**

Furthermore, the prominent cellular and molecular functions (*P* < .01) induced at 2 h post-infection of pretreated cells were Cell-To-Cell signaling and interaction, Cell Death and Survival, Cellular Development, Cellular Growth and Proliferation, and Cellular Function and Maintenance. Whereas Cell Death and Survival, Cell-To-Cell Signaling and Interaction, Cellular Movement, Cellular Function and Maintenance, and Cell Signaling were induced at 24 h post-infection of the pretreated cells. All the induced functions at 2 h and 24 h post-infection and their involved genes are listed in **Table S3.**

At 2 h post-infection, the top-scoring network connected the cellular and molecular functions were Cell-To-Cell Signaling and Interaction, Cellular Movement, Hematological System Development, and Function. The major nodes connecting the genes were the NFκB complex, CXCL1/2, CD14, IL18, and NOD2 (predicted to be activated), and MYD88, CASP1 (predicted to be inhibited). This network was linked to the CPs involved in PPRs (13 genes), TREM1 (12 genes), Inflammasome pathway (10 genes), and NF-κB signaling (7 genes) (Figure 3(e)). Whereas at 24 h post-infection, the top network was Cell Function and Maintenance, Hematological System Development and Function, Antimicrobial response. The major nodes involved were JUN, NFKBlA, NFKB1, IKBKB, RELA, IL8, IKK complex, CHUK, Ap1, p85 TCR, and P glycoprotein (predicted to be activated), and CASP8, and TRAF6 (predicted to be inhibited). This network was linked to the CPs involved in Glucocorticoid Receptor signaling (19 genes), NF-κB signaling (15 genes), IL8 (12 genes), and IL6 (12 genes) (figure 3(f)). The top networks modulated at 2 h and 24 h post-infection, score, and focus molecules are listed in Table 2.

Additionally, the top predicted transcriptional regulators (*P* < .01) at 2 h post-infection of the pre-treated cells were dextran sulfate, TLR4, kinase EIF2AK3, FCGR2A, and LEP. Whereas transcriptional regulators at 24 h were RELA, NFκB complex, MYD88, lipopolysaccharide, and IL1A. All of these regulators were predicted to be activated. The mechanistic networks associated with these transcriptional regulators at 2 h and 24 h are shown in **Fig. S1E** and **Fig. S1F**, respectively. The top upstream regulators, molecule type, activation state, overlap *p*- value, activation z-score and the target molecules are listed in Table 3.

Collectively, our results suggested that EcN exerts a protective anti-inflammatory effect on HT-29 that inhibits the proinflammatory effect caused by *C. jejuni* induced cytokines. This effect is induced through the activation of the mitogen-activated protein kinases (P38 MAPK, ERK1/2, and JNK) which play an important role in enhancing cellular tight junction integrity and epithelial barrier functions. This induction leading to activation of NFκB and AP-1, and consequently induces production of pro-inflammatory (IL1β, IL-6, IL-8, TNFα), anti-inflammatory (IL-10) cytokines, and anti-viral type 1 interferons (IFNα, IFNβ).^31^ EcN can also enhance cellular barrier integrity and protect the cells through activation of the anti-apoptotic Akt and PI3K to protect the cells from *C. jejuni* cytokine-induced apoptosis. Additionally, EcN induced glucocorticoid signaling which mediates anti-inflammatory, anti-proliferative, and immunomodulatory activity on HT-29 cells.^32^

## Discussion

Increasing concerns about antibiotic-resistance have led to the use of probiotic bacteria as an alternative intervention strategy to control foodborne pathogens.^33^ Recently, the immune-modulatory agents are used to control infectious diseases to avoid selection pressure and the evolution of microbial resistance by targeting the host rather than the pathogen.^4^ Although the previous studies corroborated that EcN’s probiotic activity might be via modulating the induction of cytokines and chemokines,^6,19,34,35^ the impact of EcN on factors that were involved in cellular immunity during *C. jejuni* infection remains uncharacterized. This is important because *C. jejuni* infection can disrupt the epithelial barrier integrity as well as stimulate the host’s pro-inflammatory response, which, in turn, might facilitate it's an invasion to the gut and cause gastroenteritis.^36–40^ Therefore, elucidating the impact of EcN on the innate responses is important to gain a mechanistic understanding of its protective effect against *C. jejuni* infections. Although numerous cell lines were used to investigate *in vitro* mechanisms, we used polarized and well-differentiated HT-29 cells due to their inherent unique properties that mimicking the *in vivo* intestinal epithelium^21–25^ and has been used previously for the evaluation of *Campylobacter* virulence^26^ and the immune-modulatory effect of the probiotic bacteria.^27^

After *C. jejuni* invasion of the cells, the intracellular bacteria have to overcome the protective immune response of host cells including inflammatory response to facilitate its survival inside the cells.^41^ In this study, we found that EcN significantly reduced the invasion of different *C. jejuni* strains to the HT-29 cells and no intracellular *C. jejuni* were recovered (Table 1; *P* < .05). To understand how EcN mediates this effect in the HT-29 cells we used Human Antibacterial Response RT2 Profiler™ PCR Arrays. Our results showed that infection of the HT-29 cells with *C. jejuni* induced different signaling pathways including TLRs, PPRs, IL-6, and NF-kB. Similar signaling pathways were induced by *C. jejuni* infection on human colonocyte cell line HCA-7.^42^ It also showed that infection of the HT-29 cells with *C. jejuni*, induced the expression of cytokines, chemokines, growth factor, and transcription regulator genes at 24 h post-infection (intracellular survival) (Figure 1; Table 3**; Table S1**). The epithelial cells recognize microbial products through TLRs (detect the extracellular microbial products), and NOD1 and NOD2 (detect intracellular microbial structures), leading to the activation of NF-κB production or interferon regulatory factors (IRF-3, 5, and 7), which consequently leads to the activation of proinflammatory gene products that recruits immune cells to the site of infection, and then initiate mechanisms for the clearance of the pathogen.^43,44^ These results were supported by previous observations that *C. jejuni* induced the expression of proinflammatory cytokines (TLR4, NOD1, IL-8, IL-1, and TNF) and chemokines (CCL2 and CCL4) through the activation of NF-κB in human epithelial monocytes and macrophage cells.^39,40,45–48^ Additionally, *C. jejuni* can activate NF-κB in the cells through the stimulation of ERK, p38 MAPK, and IL-8.^49^ The secretion of IL-8 with other pro-inflammatory cytokines is thought to contribute to the cellular processes that cause diarrhea and result in the clearance of infection. *C. jejuni* can also activate the anti-inflammatory response to benefit its survival. Our results also showed that *C. jejuni* activated ERK, p38, Akt, and P13 K, which is in agreement with previous studies.^41,49^

EcN induced IRF7, TICAM1/2, IL12A/B, IRAK1, and IFN alfa/beta, TLRs, ERK1/2, p38MAPK, Ap1, JNK, TLR4, IL1B, IL17A, and NF-κB signaling pathways and their cognate genes at 4 and 24 h post *C. jejuni* infection, which has been reported to; 1) specialize in the recognition of pathogenic bacterial products, 2) enhance barrier function by enhancing tight junction integrity, 3) produce antimicrobial peptides which impair a wide range of pathogens, 4) control maturation of dendritic cells, 5) activate production of protective pro-inflammatory and immunoregulatory chemokines and cytokines that is involved in the initiation of innate and adaptive immune responses at the infection site,^16,50–52^ and 6) enhance the epithelial-cell regeneration and control epithelial-cell apoptosis, which may enhance survival of the cells, and promote its proliferation.^53–56^ Other probiotic bacteria have been shown to modulate the immune response of the epithelial cells through their effect on the expression of innate immune genes. For example, probiotic mix, VSL#3 induced the expression of IL12, and IL10,^57^ *L. acidophilus* induced the expression of JUN, TNF, ICAMI, IL6, and IL-8,^58^ while *L. rhamnosus* GG induced the expression of IL1B, TNFA, CXCL5, CXCL2, and CCL5.^59^

Interestingly, our data suggest that the pretreatment of the intestinal cells with EcN can impact *C. jejuni*’s invasion to and survival in the HT-29 cells via modulating or “prestimulating” the epithelial cells immune defenses against *C. jejuni*. Pre-treatment of the HT-29 cells with EcN exert anti-inflammatory responses to likely counter proinflammatory response induced by *C. jejuni*. For example, the expression NF-κB, proinflammatory cytokines (IL-8, IL6, IL12B, IL18, TNF), mitogen-activated protein kinases (MAPK1, MAPK3, MAPK8, MAPK14, MAP2K3), TLRs (TLR4, TLR5), TLR adaptor molecules (TICAM1, TICAM2), apoptosis regulating genes (CASP8, RIPK2), NOD-like receptors (NOD1), ERK1/2 signaling genes (JUN), and MYD88 dependent genes (IRAK3, TRAF6) were reduced when the cells treated with EcN before *C. jejuni* infection.

Other probiotics have been reported to exert a similar effect on the cells to control bacterial infections. For example, *Lactobacillus amylovorus* induces anti-inflammatory effects through the inhibition of the proinflammatory cytokines IL-1β and IL-8 to protect the cells against the proinflammatory response induced by Enterotoxigenic *E. coli* (ETEC). This anti-inflammatory effect is mediated by the induction of negative regulators of TLR4 signaling, TOLLIP, and IRAK-M.^60^ While pretreatment of the IPEC-1 cells with *Saccharomyces cerevisiae* inhibited ETEC-induced pro-inflammatory response through the inhibition of ERK1/2 and p38 MAPK signaling pathways.^61^ In another study, *Bifidobacterium longum* and *B. breve* significantly reduced the IL-8, MCP-1, and IL-6 induction in porcine intestinal epithelial cells in response to ETEC through the modulation of NFκB and MAPK pathways.^62^ *Lactobacillus jensenii* and *L. casei* inhibited TLR4-dependent NFκB and MAPK activation, leading to reduction of proinflammatory cytokines and chemokines expression caused by ETEC infection. These probiotics modulated their effect via inhibition of NFκB and p38 MAPK activation in epithelial cells which then reduced the expression of IL-6, IL-8, IL-1β, and MCP-1.^63,64^ Furthermore, *L. johnsonii* and VSL#3 alleviated the induction of IL6, IL10, NOD, and TNF caused by *C. jejuni* in a mouse model that mimic *C. jejuni* induced immunopathology in humans.^54,65^

Probiotics have been shown to confer protection against many cellular stresses including apoptosis.^66^ Similarly, EcN may protect the HT-29 cells by the activation of the anti-apoptotic Akt and PI3K genes. In another study, LGG enhanced membrane barrier integrity and protective responses through the activation of the anti-apoptotic PKB/Akt in a PI3K-dependent manner to protect the cells from cytokine-induced apoptosis.^55^ The ability of EcN to regulate cellular apoptosis might be a useful strategy for the prevention of reduced membrane integrity caused by *C. jejuni*. Therefore, we suggest that EcN’s negates *C. jejuni* not only by enhancing the intestinal barrier strength^20^ but also via modulating the immune response, likely toward a preactivated (primed) steady-state and activation of the anti-apoptotic-associated genes. This was supported by previous observations that EcN can prime the immune response of the intestinal cells by affecting the expression of several cytokines and chemokines to control bacterial invasion and survival.^6,19,34,35^ Thus, our results further highlight immune-modulating probiotic properties of EcN and demonstrate its use for the control of *C. jejuni*.

In this study, we expanded our understanding of the immunomodulatory mechanisms via which EcN can impact *C. jejuni* infection of human intestinal cells. In light of the absence of commercially available anti-*Campylobacter* vaccines and the potential increase in antibiotic-resistant infections, alternative methods to combat *C. jejuni* are needed. Our study may facilitate the use of probiotic EcN as a potential antibiotic-independent approach to control *C. jejuni* infections in humans and poultry.

## Material and methods

### Cell line, bacterial strains and their growth conditions

The polarized human colorectal adenocarcinoma cells (HT-29; ATCC HTB-38) were cultured and maintained as described before.^20^ We have selected the HT-29 cells in particular as an infection model *in vitro* because it is polarized and a well-differentiated cell line. The polarized HT-29 cells are characterized by the presence of apical brush border proteins, presence of Cl-channels and Cl-secretion, mucus production, expression of disaccharides and peptidase enzymes, the formation of domes on impermeable substrates, display of trans-epithelial resistance typical of polarized epithelium and intracellular tight junction proteins which makes the cells closely mimicking the *in vivo* functional intestinal epithelium.^21–25^ The *C. jejuni* strains were isolated from different hosts; human (811–67, 81116 and NCTC11168), poultry (chicken 1, chicken 2, chicken 3, chicken 4, and chicken 5),^67^ turkey (LSE-30, LSW-66, and BOS-114),^68^ beef cattle (Cj-N-33, Cj-M-48, Cj-M-64),^69^ dairy cattle (G-D-20, G-D-5, and G-D-14),^70^ and starlings (I-Br-14, E-Br-12, and F-Br-1)^70^ (Table 1) and were used to test the adhesion, invasion and survival of *C. jejuni* in polarized HT-29 cells. *C. jejuni* strains were grown using Mueller-Hinton agar (MH; Difco) supplemented with a *Campylobacter* selective supplement (CSS; SR0117; Oxoid) at 42°C under microaerobic conditions (5% O_2_, 10% CO_2_, and 85% N_2_). EcN was grown aerobically to logarithmic phase using Luria-Bertani (LB; Difco) broth at 37°C.

### Gentamycin protection assay

The effect of EcN on *C. jejuni*’s invasion to and survival in polarized HT-29 cells was evaluated using gentamycin protection assay as described previously.^20^ EcN grown to logarithmic phase was pelleted, washed with Dulbecco’s phosphate-buffered saline (DPBS), and re-suspended in Dulbecco’s Modified Eagle’s Medium (DMEM). About 100 µl (1 × 10^7^ CFU) was added to each well of a 96 well plate containing the HT-29 monolayers, and the plate was incubated for 4 h. The HT-29 cells were washed to remove the extracellular bacteria and then infected with 1.7 × 10^7^ CFUs of *C. jejuni* for 2 h. For adhesion, the *Campylobacter* infected HT-29 cells were washed 3 times with PBS and the adherent *C. jejuni* CFUs were enumerated after lysis with 0.1% Triton X-100, 10-fold serial dilution, and plating onto MH agar plates containing CSS. To evaluate the effect of EcN on *C. jejuni*’s invasion to the HT-29 cells, at 2 h post-infection, the HT-29 cells were washed and treated with DMEM containing 150 µg/ml gentamicin for 1 h. The cells were then washed and *C. jejuni* CFUs was enumerated after lysis with 0.1% Triton X-100, 10-fold serial dilution, and plating onto MH agar plates containing CSS. To evaluate the effect of EcN on *C. jejuni*’s intracellular survival, following the gentamicin treatment, the HT-29 cells were washed and incubated for 24 h in fresh DMEM containing 10 µg/ml gentamicin. The number of internal *C. jejuni* was determined as above.

### Human antibacterial response RT2 profiler™ PCR arrays

The expression of 84 innate immune-associated genes was determined using Human Antibacterial Response RT2 Profiler™ PCR Arrays (Qiagen, Array # PAHS-148Z).^71^ Polarized HT-29 cells were treated with EcN for 4 h and then infected with *C. jejuni* for 2 h (invasion) and 24 h (intracellular survival). Following treatment, the HT-29 cells were washed and suspended in the TRIzol reagent (Life technologies). Untreated HT-29 cells, HT-29 cells treated with EcN, and HT-29 cells treated with *C. jejuni* alone were used as controls. Total RNA was extracted from the HT-29 cells using the miRNeasy Mini Kit (Qiagen) and traces of DNA was removed using the Qiagen RT^2^ First Strand Kit (Qiagen) as described by the manufacturer. The cDNA was synthesized using the RT^2^ First strand kit and analyzed using RT^2^ Profiler™ PCR Arrays. Changes in gene expression were determined using the ∆∆Ct method.^72^ Significantly altered genes were further analyzed using the IPA software (www.ingenuity.com) to identify innate pathways, key regulators and their activities, detect cellular and molecular functions, and to predict downstream effects on biological processes modulated in HT-29 cells in response to EcN, *C. jejuni*, and EcN+ *C. jejuni*. The experiment was repeated 2 times with duplicate samples in each experiment.

## Statistical analysis

Data for adherence, invasion, and intracellular survival of *C. jejuni* were presented as a percentage of inhibition in comparison to the non-treated control. Two-way analysis of variance (ANOVA) was used to analyze the qPCR data. A fold change of ± 1.5 ⩾ or ⩽ 1.5 and a *P*-value ≤0.05 was used to determine significant differences in the expression of the genes. Significantly modulated CP was calculated via a right-tailed Fisher’s Exact test using a -log (*p*-value) cutoff of 1.3 at *P*< .05.

## Supplementary Material

Supplemental Material
