# Supplementary material for: Immuno-modulatory effect of probiotic E. coli Nissle 1917 in polarized human colonic cells against Campylobacter jejuni infection
Source: Gut Microbes. 2020 Dec 31;13(1):1857514. doi: 10.1080/19490976.2020.1857514 (PMC7781529; doi:10.1080/19490976.2020.1857514)
Supplement: Supplemental Material [file KGMI_A_1857514_SM8366.zip › Supplementary information/Supplementary tables final.docx]

**Supplementary table 1:** Fold change in the expression of innate immune response genes in response to different treatments at 2 h and 24 h post-infection. The up-regulated and down-regulated genes were determined using a cutoff ± ≥ 1.5 or ≤ 1.5 and a P ≤ 0.05.

| **Gene** | **Gene product description** | **Fold change** | | | | | |
| --- | --- | --- | --- | --- | --- | --- | --- |
|  |  | **2h post-infection** | | | **24h post-infection** | | |
|  |  | **EcN** | **Cj** | **EcN+ Cj** | **EcN** | **Cj** | **EcN+ Cj** |
| AKT1 | V-akt murine thymoma viral oncogene homolog 1 | 2.6 | - | - | 2.8 | 2.0 | 4.9 |
| BIRC3 | Baculoviral IAP repeat containing 3 | -1.8 | 3.2 | 4.2 | 3.4 | 9.3 | 15.5 |
| BPI | Bactericidal/permeability-increasing protein | - | -4.4 | -5.9 | - | - | - |
| CAMP | Cathelicidin antimicrobial peptide | 2.0 | 2.4 | -2.2 | -1.6 | 1.7 | 3.1 |
| CARD6 | Caspase recruitment domain family, member 6 | 1.8 | - | -1.5 | 2.3 | - | 2.4 |
| CARD9 | Caspase recruitment domain family, member 9 | 1.8 | - | - | 2.1 | 2.6 | 5.2 |
| CASP1 | Caspase 1, apoptosis-related cysteine peptidase (interleukin 1, beta, convertase) | -2.7 | 2.0 | -2.7 | - | -1.8 | 3.1 |
| CASP8 | Caspase 8, apoptosis-related cysteine peptidase | 2.4 | 1.8 | - | - | 4.7 | -1.7 |
| CCL5 | Chemokine (C-C motif) ligand 5 | -3.0 | -1.5 | -7.1 | 1.7 | -4.8 | -4.3 |
| CD14 | CD14 molecule | 2.8 | - | 4.3 | 1.9 | -1.6 | 4.0 |
| CHUK | Conserved helix-loop-helix ubiquitous kinase | 1.5 | 1.6 | -1.5 | - | 2.0 | 1.5 |
| CXCL1 | Chemokine (C-X-C motif) ligand 1 (melanoma growth stimulating activity, alpha) | -1.9 | 1.7 | 3.4 | 30.3 | 18.5 | 7.1 |
| CXCL2 | Chemokine (C-X-C motif) ligand 2 | -1.9 | 1.7 | 2.8 | 21.0 | 13.6 | 7.4 |
| DMBT1 | Deleted in malignant brain tumors 1 | - | - | - | 22.6 | - | - |
| FADD | Fas (TNFRSF6)-associated via death domain | -1.5 | 2.2 | -1.7 | - | - | 2.2 |
| HSP90AA1 | Heat shock protein 90kDa alpha (cytosolic), class A member 1 | - | 1.5 | 2.2 | 1.6 | 3.2 | 2.0 |
| IFNA1 | Interferon, alpha 1 | -2.5 | -1.8 | -2.6 | 1.7 | 1.6 | -1.6 |
| IFNB1 | Interferon, beta1, fibroblast | -6.9 |  | -7.2 | 4.5 |  | -1.5 |
| IKBKB | Inhibitor of kappa light polypeptide gene enhancer in B-cells, kinase beta | 8.7 | 2.4 | - | 2.1 | 2.5 | 3.3 |
| IL12A | Interleukin 12A (natural killer cell stimulatory factor 1, cytotoxic lymphocyte maturation factor 1) | -2.4 | 2.0 | 3.3 | 3.8 | 18.9 | 1.7 |
| IL12B | Interleukin 12B (natural killer cell stimulatory factor 2, cytotoxic lymphocyte maturation factor 2) | - | - | - | 3.7 | 5.6 | -26.5 |
| IL18 | Interleukin 18 (interferon-gamma-inducing factor) | 2.5 | - | 1.8 | - | 4.6 | 2.5 |
| IL1B | Interleuking 1, beta | 1.5 | -2.3 | 6.0 | 2.2 |  | 8.3 |
| IL6 | Interleukin 6 (interferon, beta 2) | - | - | - | 3.3 | 9.7 | 2.3 |
| CXCL8 | Interleukin 8 | -4.6 | -1.6 | -4.7 | 10.4 | 7.8 | 1.9 |
| IRAK1 | Interleukin-1 receptor-associated kinase 1 | 2.1 | 3.9 | - | 2.7 | 1.7 | 3.8 |
| IRAK3 | Interleukin-1 receptor-associated kinase 3 | 15.6 | 3.7 | 12.6 | 1.6 | 4.0 | -2.5 |
| IRF5 | Interferon regulatory factor 5 | 5.7 | 2.8 | 4.3 | 2.9 | 4.5 | 6.8 |
| IRF7 | Interferon regulatory factor 7 | -1.6 | -1.7 | - | 3.2 | 5.5 | 5.2 |
| JUN | Jun proto-oncogene | 6.6 | -3.6 | 4.4 | 9.8 | 27.6 | 12.2 |
| LBP | Lipopolysaccharide binding protein | 23.8 | 8.7 | - | - | -2.5 | - |
| LCN2 | Lipocalin 2 | - | 1.9 | - | -1.8 | 2.5 | -3.6 |
| LTF | Lactotransferrin | - | 1.7 | - | - | 8.9 | -2.2 |
| LY96 | Lymphocyte antigen 96 | - | 1.6 | - | 1.6 | 2.0 | 7.1 |
| LYZ | Lysozyme | 2.2 | 1.6 | - | - | - | 1.5 |
| MAP2K1 | Mitogen-activated protein kinase 1 | 2.8 | 1.9 | 2.5 | 2.1 | 1.7 | 1.5 |
| MAP2K3 | Mitogen-activated protein kinase 3 | 1.9 | - | 1.6 | 2.3 | 7.6 | -1.6 |
| MAP2K4 | Mitogen-activated protein kinase 4 | 1.6 | 1.8 | - | - | 2.8 | 1.8 |
| MAP3K7 | Mitogen-activated protein kinase 7 | 1.9 | 1.6 | - | 1.5 | - | - |
| MAPK1 | Mitogen-activated protein kinase 1 | 2.5 | 2.0 | - | - | - | -1.5 |
| MAPK14 | Mitogen-activated protein kinase 14 | 4.2 | 3.4 | 3.1 | 1.6 | 2.5 | 1.9 |
| MAPK3 | Mitogen-activated protein kinase 3 | 3.9 | 4.1 | 4.6 | 1.7 | 2.3 | -1.5 |
| MAPK8 | Mitogen-activated protein kinase 8 | 1.9 | 2.4 | -1.7 | - | 3.7 | -1.5 |
| MYD88 | Myeloid differentiation primary response gene (88) | -1.5 | -2.0 | -2.0 | 1.5 | - | -1.5 |
| NAIP | MLR family, apoptosis inhibitory protein | 2.6 | - | 1.7 | - | - | 1.5 |
| NFKB1 | Nuclear factor of kappa light polypeptide gene enhancer in B-cells 1 | 4.1 | 3.0 | 2.7 | - | 2.2 | 1.5 |
| NFKBIA | Nuclear factor of kappa light polypeptide gene enhancer in B-cells inhibitor, alpha | -2.8 | -1.5 | -1.6 | 2.8 | 4.1 | 2.5 |
| NLRC4 | NLR family, CARD domain containing 4 | 1.6 | 1.8 | - | 1.4 | 2.8 | 2.0 |
| NLRP1 | NLR family, CARD domain containing 1 | - | - | - | - | - | 8.3 |
| NLRP3 | NLR family, CARD domain containing 3 | 2.5 | -1.6 | - | 1.9 | 1.7 | - |
| NOD1 | Nucleotide-binding oligomerization domain containing 1 | 1.5 | 1.6 | -1.6 | 2.5 | 3.5 | 1.9 |
| NOD2 | Nucleotide-binding oligomerization domain containing 2 | - | - | 1.9 | - | 2.1 | 1.8 |
| PIK3CA | Phosphoinositide-3-kinase, catalytic, alpha polypeptide | 1.6 | 2.2 | - | - | 4.1 | 3.0 |
| PRTN3 | Proteinase 3 | - | - | -4.1 | 2.7 | - | 2.0 |
| PSTPIP1 | Proline-serine-threonine phosphatase interacting protein 1 | -2.0 | 1.8 | -1.6 | - | -1.8 | -1.6 |
| PYCARD | PYD and CARD domain containing | 1.5 | 2.0 | - | - | - | - |
| RAC1 | Ras-related C3 botulinum toxin substrate 1 (rho family, small GTP binding protein Rac1) | 2.0 | 1.6 | 1.6 | - | - | -1.8 |
| RELA | V-rel reticulendotheliosis viral oncogene homolog A (avian) | 3.9 | 2.1 | 1.5 | 3.3 | 6.2 | 7.5 |
| RIPK1 | Receptor (TNFRSF)-interacting serine-threonine kinase 1 | - | - | -2.0 | 2.1 | 3.1 | 2.1 |
| RIPK2 | Receptor-interacting serine-threonine kinase 2 | -1.7 | 1.7 | - | - | 4.1 | -1.7 |
| SLC11A1 | Solute carrier family 11 (metal ion transporters), member 1 | 22.0 | 3.7 | 7.3 | - | 2.8 | 2.2 |
| SLPI | Secretory leukocyte peptidase inhibitor | - | 2.1 | - | - | 3.6 | 2.7 |
| SUGT1 | SGT1, suppressor of G2 allele of SKP1 | - | - | 1.7 | - | 4.5 | -2.1 |
| TICAM1 | Toll-like receptor adaptor molecule 1 | 1.5 | - | - | 1.8 | 11.1 | 8.0 |
| TICAM2 | Toll-like receptor adaptor molecule 2 | -1.8 | -2.0 | -1.7 | 1.9 | 8.4 | 3.7 |
| TIRAP | Toll-interleukin 1 receptor | 3.7 | 4.3 | 11.2 | 2.0 | 3.1 | 2.9 |
| TLR1 | Toll-like receptor 1 | -4.1 | -3.1 | -6.3 | 4.4 | 4.2 | 7.1 |
| TLR4 | Toll-like receptor 4 | - | 1.7 | 2.5 | 1.5 | 4.3 | -3.6 |
| TLR5 | Toll-like receptor 5 | -1.5 | - | 2.0 | 12.8 | 9.4 | 3.3 |
| TLR6 | Toll-like receptor 6 | 1.5 | 1.7 | -1.8 | 1.6 | 1.6 | -2.0 |
| TNF | Tumor Necrosis Factor | 2.8 | 2.1 | 1.8 | 24.1 | 44.9 | 23.8 |
| TNFRSF1A | Tumor cecrosis factor receptor superfamily, member A1 | 3.2 | 3.0 | 1.5 | 2.3 | 4.2 | 3.9 |
| TOLLIP | Toll interacting protein | 2.6 | - | 2.9 | 2.2 | 5.6 | 5.0 |
| TRAF6 | TNF receptor-associated factor 6 | 2.6 | 2.0 | 1.8 | 1.7 | 7.4 | -1.5 |
| XIAP | X-linked inhibitor of apoptosis | 2.2 | 2.6 | - | - | 6.3 | 3.5 |
| ZBP1 | Z-DNA binding protein 1 | 4.1 | 3.4 | 2.7 | 4.1 | - | -3.6 |

(-) not significantly regulated

**Supplementary table 2:** The top significantly modulated canonical pathways in HT-29 cells with and without EcN and *C. jejuni*.

| **Canonical pathway** | **-log(p-value)** | **Ratio** | **Molecules** |
| --- | --- | --- | --- |
| **EcN/ 4 h post- treatment** | | | |
| Toll-like Receptor Signaling | 59 | 0.40 | JUN,MAPK14,TLR5,IL12A,MYD88,TLR1,TICAM1,TIRAP,LBP,NFKBIA,IRAK3,LY96,TNF,TOLLIP,MAP2K3,MAPK8,TLR6,TRAF6,IRAK1,MAP3K7,NFKB1,CHUK,IL1B,CD14,IKBKB,RELA,IL18,MAP2K4,MAPK1,TICAM2 |
| Neuroinflammation Signaling Pathway | 54.3 | 0.12 | JUN,MAPK14,TLR5,IL12A,AKT1,MYD88,TNFRSF1A,TLR1,TICAM1,TIRAP,IRAK3,BIRC3,IRF7,PIK3CA,TNF,XIAP,MAPK8,TLR6,MAPK3,TRAF6,IRAK1,NFKB1,CHUK,IL1B,CCL5,CASP1,IKBKB,PYCARD,RELA,CXCL8,IL18,MAP2K4,NLRP3,IFNA1/IFNA13,MAPK1,TICAM2,IFNB1,CASP8 |
| Role of Pattern Recognition Receptors in Recognition of Bacteria and Viruses (PPRs) | 43.3 | 0.20 | TLR5,IL12A,MYD88,TLR1,TICAM1,NOD1,IRF7,PIK3CA,TNF,RIPK2,MAPK8,TLR6,MAPK3,TRAF6,NFKB1,IL1B,CCL5,CASP1,RELA,CXCL8,IL18,MAP2K4,NLRP3,IFNA1/IFNA13,MAPK1,IFNB1,NLRC4 |
| IL-6 Signaling | 37.7 | 0.19 | JUN,MAP3K7,MAPK14,NFKB1,CHUK,AKT1,IL1B,TNFRSF1A,CD14,IKBKB,LBP,MAP2K1,NFKBIA,RELA,CXCL8,IL18,PIK3CA,TNF,MAP2K4,MAPK1,MAP2K3,MAPK8,MAPK3,TRAF6 |
| Acute Phase Response Signaling | 34.5 | 0.14 | JUN,IRAK1,MAP3K7,MAPK14,NFKB1,CHUK,AKT1,IL1B,MYD88,TNFRSF1A,IKBKB,LBP,MAP2K1,NFKBIA,RELA,IL18,PIK3CA,TNF,MAP2K4,MAPK1,MAP2K3,MAPK8,MAPK3,TRAF6 |
| **EcN/ 24 h post- treatment** | | | |
| Toll-like Receptor Signaling | 50.5 | 0.34 | JUN,TLR5,MAPK14,IL12A,TLR1,TICAM1,TIRAP,NFKBIA,IRAK3,LY96,TNF,TOLLIP,MAP2K3,IL12B,TLR6,MAPK8,TRAF6,IRAK1,MAP3K7,IL1B,CD14,IKBKB,RELA,MAP2K4,MAPK1,TICAM2 |
| Neuroinflammation Signaling Pathway | 48.8 | 0.11 | JUN,TLR5,MAPK14,IL12A,AKT1,TNFRSF1A,TLR1,TICAM1,TIRAP,IL6,IRAK3,BIRC3,IRF7,PIK3CA,TNF,IL12B,TLR6,MAPK8,RIPK1,MAPK3,TRAF6,IRAK1,IL1B,CCL5,IKBKB,PYCARD,CXCL8,RELA,IFNA1/IFNA13,MAP2K4,NLRP3,MAPK1,TICAM2,IFNB1 |
| PPRs | 38.5 | 0.18 | TLR5,IL12A,IL1B,CCL5,TLR1,TICAM1,NOD1,IL6,CXCL8,RELA,IRF7,PIK3CA,TNF,IFNA1/IFNA13,MAP2K4,NLRP3,MAPK1,IL12B,TLR6,MAPK8,IFNB1,NLRC4,MAPK3,TRAF6 |
| IL-6 Signaling | 32.8 | 0.16 | JUN,MAP3K7,MAPK14,AKT1,IL1B,TNFRSF1A,CD14,IKBKB,MAP2K1,IL6,NFKBIA,CXCL8,RELA,PIK3CA,TNF,MAP2K4,MAPK1,MAP2K3,MAPK8,MAPK3,TRAF6 |
| Acute Phase Response Signaling | 30.0 | 0.12 | JUN,IRAK1,MAP3K7,MAPK14,AKT1,IL1B,TNFRSF1A,IKBKB,MAP2K1,IL6,NFKBIA,RELA,PIK3CA,TNF,MAP2K4,MAPK1,MAP2K3,MAPK8,RIPK1,MAPK3,TRAF6 |
| ***C. jejuni/* 2 h post- infection** | | | |
| Neuroinflammation Signaling Pathway | 52.3 | 0.12 | JUN,MAPK14,IL12A,MYD88,AKT1,TNFRSF1A,TLR1,TIRAP,IRAK3,BIRC3,TLR4,IRF7,PIK3CA,TNF,XIAP,MAPK8,TLR6,TRAF6,MAPK3,IRAK1,NFKB1,CHUK,IL1B,CCL5,CASP1,IKBKB,PYCARD,RELA,CXCL8,IL18,MAP2K4,IFNA1/IFNA13,NLRP3,MAPK1,TICAM2,IFNB1,CASP8 |
| Toll-like Receptor Signaling | 48.8 | 0.34 | JUN,MAPK14,IL12A,MYD88,TLR1,TIRAP,LBP,NFKBIA,IRAK3,TLR4,LY96,TNF,MAPK8,TLR6,TRAF6,IRAK1,MAP3K7,NFKB1,CHUK,IL1B,IKBKB,RELA,IL18,MAP2K4,MAPK1,TICAM2 |
| PPRs | 41.1 | 0.19 | IL12A,MYD88,TLR1,NOD1,TLR4,IRF7,PIK3CA,TNF,RIPK2,MAPK8,TLR6,TRAF6,MAPK3,NFKB1,IL1B,CCL5,CASP1,RELA,CXCL8,IL18,MAP2K4,IFNA1/IFNA13,NLRP3,MAPK1,IFNB1,NLRC4 |
| IL-6 Signaling | 33.5 | 0.17 | JUN,MAP3K7,MAPK14,NFKB1,CHUK,IL1B,AKT1,TNFRSF1A,IKBKB,LBP,MAP2K1,NFKBIA,RELA,CXCL8,IL18,PIK3CA,MAP2K4,TNF,MAPK1,MAPK8,TRAF6,MAPK3 |
| NF-kB Signaling | 31.9 | 0.13 | IRAK1,MAP3K7,NFKB1,CHUK,MYD88,IL1B,AKT1,TNFRSF1A,TLR1,TIRAP,IKBKB,FADD,NFKBIA,IRAK3,RELA,TLR4,IL18,PIK3CA,TNF,MAPK8,TLR6,CASP8,TRAF6 |
| ***C. jejuni/* 24 h post- infection** | | | |
| Toll-like Receptor Signaling | 43.2 | 0.30 | JUN,IRAK1,MAPK14,NFKB1,TLR5,CHUK,IL12A,IL1B,TLR1,CD14,TICAM1,TIRAP,IKBKB,LBP,NFKBIA,RELA,TLR4,LY96,TNF,TOLLIP,TICAM2,MAP2K3,IL12B |
| Neuroinflammation Signaling Pathway | 41 | 0.10 | JUN,MAPK14,TLR5,IL12A,AKT1,TNFRSF1A,TLR1,TICAM1,TIRAP,BIRC3,TLR4,IRF7,PIK3CA,TNF,XIAP,IL12B,RIPK1,IRAK1,NFKB1,CHUK,IL1B,CCL5,CASP1,IKBKB,CXCL8,RELA,IFNA1/IFNA13,NLRP3,TICAM2,CASP8 |
| PPRs | 32.3 | 0.15 | NFKB1,TLR5,IL12A,IL1B,CCL5,TLR1,NOD2,TICAM1,NOD1,CASP1,CXCL8,RELA,TLR4,IRF7,PIK3CA,TNF,IFNA1/IFNA13,NLRP3,RIPK2,IL12B,NLRC4 |
| IL-6 Signaling | 24.8 | 0.13 | JUN,MAPK14,NFKB1,CHUK,AKT1,IL1B,TNFRSF1A,CD14,IKBKB,LBP,MAP2K1,NFKBIA,CXCL8,RELA,PIK3CA,TNF,MAP2K3 |
| TREM1 Signaling | 24.7 | 0.20 | IRAK1,NFKB1,TLR5,AKT1,IL1B,TLR1,NOD2,NOD1,CASP1,CXCL8,RELA,TLR4,TNF,NLRP3,NLRC4 |
| ***EcN+ C. jejuni/* 2 h post- infection** | | | |
| Neuroinflammation Signaling Pathway | 46 | 0.11 | JUN,TLR5,MAPK14,IL12A,AKT1,MYD88,TNFRSF1A,TLR1,TIRAP,IRAK3,BIRC3,TLR4,IRF7,TNF,XIAP,MAPK8,TLR6,RIPK1,MAPK3,TRAF6,NFKB1,CHUK,IL1B,CCL5,CASP1,RELA,PYCARD,CXCL8,IL18,IFNA1/IFNA13,MAPK1,TICAM2,IFNB1 |
| Toll-like Receptor Signaling | 44.9 | 0.32 | JUN,TLR5,MAPK14,NFKB1,IL12A,CHUK,IL1B,MYD88,CD14,TLR1,TIRAP,NFKBIA,IRAK3,RELA,TLR4,IL18,TNF,MAPK1,TOLLIP,TICAM2,MAP2K3,MAPK8,TLR6,TRAF6 |
| PPRs | 35.9 | 0.17 | TLR5,NFKB1,IL12A,IL1B,MYD88,CCL5,NOD2,TLR1,NOD1,CASP1,RELA,CXCL8,TLR4,IL18,IRF7,TNF,IFNA1/IFNA13,MAPK1,MAPK8,TLR6,IFNB1,MAPK3,TRAF6 |
| IL-6 Signaling | 28.3 | 0.14 | JUN,MAPK14,NFKB1,CHUK,AKT1,IL1B,CD14,TNFRSF1A,MAP2K1,NFKBIA,RELA,CXCL8,IL18,TNF,MAPK1,MAP2K3,MAPK8,MAPK3,TRAF6 |
| NF-kB Signaling | 27.1 | 0.11 | TLR5,NFKB1,CHUK,AKT1,IL1B,MYD88,TNFRSF1A,TLR1,TIRAP,FADD,NFKBIA,IRAK3,RELA,TLR4,IL18,TNF,MAPK8,TLR6,RIPK1,TRAF6 |
| ***EcN+ C. jejuni/* 24 h post- infection** | | | |
| Toll-like Receptor Signaling | 56.7 | 0.40 | JUN,TLR5,MAPK14,IL12A,MYD88,TLR1,TICAM1,TIRAP,LBP,NFKBIA,IRAK3,TLR4,LY96,TNF,TOLLIP,MAP2K3,IL12B,MAPK8,TLR6,TRAF6,IRAK1,NFKB1,CHUK,IL1B,CD14,IKBKB,RELA,MAP2K4,TICAM2,MAPK1 |
| Neuroinflammation Signaling Pathway | 53.1 | 0.13 | JUN,TLR5,MAPK14,IL12A,AKT1,MYD88,TNFRSF1A,TLR1,TICAM1,TIRAP,IL6,BIRC3,IRAK3,TLR4,IRF7,PIK3CA,TNF,XIAP,IL12B,MAPK8,TLR6,RIPK1,MAPK3,TRAF6,IRAK1,NFKB1,CHUK,IL1B,CCL5,CASP1,IKBKB,CXCL8,RELA,IFNA1/IFNA13,MAP2K4,TICAM2,MAPK1,IFNB1,CASP8 |
| PPRs | 45.5 | 0.21 | TLR5,IL12A,MYD88,TLR1,TICAM1,NOD1,IL6,TLR4,IRF7,PIK3CA,TNF,RIPK2,IL12B,MAPK8,TLR6,MAPK3,TRAF6,NFKB1,IL1B,CCL5,NOD2,CASP1,CXCL8,RELA,IFNA1/IFNA13,MAP2K4,MAPK1,IFNB1,NLRC4 |
| IL-6 Signaling | 33.9 | 0.18 | JUN,MAPK14,NFKB1,CHUK,IL1B,AKT1,TNFRSF1A,CD14,IKBKB,LBP,MAP2K1,IL6,NFKBIA,CXCL8,RELA,PIK3CA,TNF,MAP2K4,MAPK1,MAP2K3,MAPK8,MAPK3,TRAF6 |
| TREM1 Signaling | 24.7 | 0.20 |  |

The ratio is calculated by dividing the number of genes from the data set of differentially expressed genes that participate in a canonical pathway by the total number of genes in that canonical pathway in IPA. The significance values of the canonical pathways was calculated by Fisher's exact test right-tailed using a -log (p-value) cutoff of 1.3, meaning that pathways with a p-value ≥ 0.05.

**Supplementary table 3:** The top cellular and molecular functions of differentially expressed genes in HT-29 cells following treatment with EcN,

*C.* *jejuni* and EcN*+ C.* *jejuni*

| **Category** | **p-value overlap** | **Molecules** |
| --- | --- | --- |
| **EcN/ 4 h post- treatment** | | |
| Cell-To-Cell Signaling and Interaction | 1.99E-50-2.12E-11 | TLR5,IL12A,TLR1,TNFRSF1A,TIRAP,TICAM1,NOD1,CARD9,MAP2K1,FADD,NFKBIA,BIRC3,CXCL1,RIPK2,SLC11A1,TRAF6,IL1B,CCL5,CD14,CASP1,IKBKB,CTSG,PSTPIP1,CXCL8,LYZ,IL18,ZBP1,JUN,MAPK14,MYD88,AKT1,LBP,CAMP,IRAK3,LY96,IRF7,PIK3CA,TNF,TOLLIP,MAP2K3,MAPK8,TLR6,HSP90AA1,MAPK3,IRAK1,MAP3K7,NFKB1,CHUK,IRF5,PYCARD,RELA,MAP2K4,NLRP3,IFNA1/IFNA13,MAPK1,IFNB1,RAC1,CASP8 |
| Cell Death and Survival | 2.8E-47-1.02E-11 | IL12A,TNFRSF1A,TLR1,TICAM1,NOD1,TIRAP,CARD9,FADD,MAP2K1,NFKBIA,BIRC3,CXCL1,XIAP,RIPK2,SLC11A1,TRAF6,IL1B,CCL5,CD14,CASP1,IKBKB,CTSG,CXCL8,LYZ,IL18,TICAM2,JUN,MAPK14,AKT1,MYD88,LBP,CAMP,IRAK3,IRF7,PIK3CA,TNF,TOLLIP,MAP2K3,MAPK8,TLR6,HSP90AA1,MAPK3,IRAK1,MAP3K7,NFKB1,CHUK,IRF5,RELA,PYCARD,NAIP,NLRP3,MAP2K4,IFNA1/IFNA13,MAPK1,IFNB1,RAC1,CASP8,NLRC4 |
| Cellular Development | 4.92E-43-1.25E-11 | TLR5,IL12A,TNFRSF1A,TLR1,NOD1,TICAM1,TIRAP,CARD9,FADD,MAP2K1,NFKBIA,BIRC3,CXCL1,RIPK2,XIAP,TRAF6,CCL5,IL1B,CD14,CASP1,IKBKB,CTSG,PSTPIP1,CXCL8,IL18,TICAM2,JUN,MAPK14,AKT1,MYD88,CAMP,IRAK3,LY96,IRF7,PIK3CA,TNF,MAP2K3,MAPK8,TLR6,HSP90AA1,MAPK3,IRAK1,MAP3K7,NFKB1,CHUK,IRF5,RELA,PYCARD,MAP2K4,IFNA1/IFNA13,NLRP3,MAPK1,IFNB1,RAC1,CASP8,NLRC4 |
| Cellular Growth and Proliferation | 4.92E-43-1.25E-11 | TLR5,IL12A,TNFRSF1A,TLR1,TICAM1,TIRAP,CARD9,FADD,MAP2K1,NFKBIA,BIRC3,CXCL1,RIPK2,XIAP,TRAF6,CCL5,IL1B,CD14,CASP1,IKBKB,CTSG,PSTPIP1,CXCL8,LYZ,IL18,TICAM2,JUN,MAPK14,AKT1,MYD88,CAMP,IRAK3,LY96,IRF7,PIK3CA,TNF,MAP2K3,MAPK8,TLR6,HSP90AA1,MAPK3,IRAK1,MAP3K7,NFKB1,CHUK,IRF5,RELA,PYCARD,MAP2K4,IFNA1/IFNA13,MAPK1,IFNB1,RAC1,CASP8,NLRC4 |
| Cellular Function and Maintenance | 5.26E-41-1.09E-11 | TLR5,IL12A,TLR1,TNFRSF1A,TICAM1,NOD1,TIRAP,CARD9,FADD,MAP2K1,NFKBIA,BIRC3,CXCL1,RIPK2,XIAP,SLC11A1,TRAF6,CCL5,IL1B,CD14,CASP1,IKBKB,CTSG,PSTPIP1,CXCL8,IL18,TICAM2,JUN,MAPK14,MYD88,AKT1,CAMP,IRAK3,LY96,IRF7,PIK3CA,TNF,MAP2K3,MAPK8,TLR6,HSP90AA1,MAPK3,IRAK1,MAP3K7,NFKB1,CHUK,IRF5,PYCARD,RELA,MAP2K4,IFNA1/IFNA13,NLRP3,MAPK1,IFNB1,RAC1,NLRC4,CASP8 |
| **EcN/ 24 h post- treatment** | | |
| Cell Death and Survival | 1.74E-43-3.64E-10 | IL12A,TNFRSF1A,TLR1,TICAM1,NOD1,TIRAP,CARD9,MAP2K1,NFKBIA,BIRC3,CXCL1,IL12B,RIPK1,TRAF6,CCL5,IL1B,CD14,IKBKB,LCN2,CXCL8,LYZ,PRTN3,TICAM2,JUN,MAPK14,AKT1,IL6,IRAK3,IRF7,PIK3CA,TNF,TOLLIP,MAP2K3,MAPK8,TLR6,HSP90AA1,MAPK3,IRAK1,MAP3K7,CXCL2,IRF5,RELA,PYCARD,NLRP3,MAP2K4,IFNA1/IFNA13,MAPK1,IFNB1,NLRC4 |
| Cell-To-Cell Signaling and Interaction | 6.96E-41-3.65E-10 | TLR5,IL12A,TLR1,TNFRSF1A,TIRAP,TICAM1,NOD1,DMBT1,CARD9,MAP2K1,NFKBIA,BIRC3,CXCL1,IL12B,RIPK1,TRAF6,IL1B,CCL5,CD14,IKBKB,LCN2,CXCL8,LYZ,PRTN3,ZBP1,JUN,MAPK14,AKT1,IL6,IRAK3,LY96,IRF7,PIK3CA,TNF,TOLLIP,MAP2K3,MAPK8,TLR6,HSP90AA1,MAPK3,IRAK1,MAP3K7,CXCL2,IRF5,PYCARD,RELA,MAP2K4,NLRP3,IFNA1/IFNA13,MAPK1,IFNB1 |
| Cellular Function and Maintenance | 8.25E-37-2.65E-10 | TLR5,IL12A,TLR1,TNFRSF1A,TICAM1,NOD1,TIRAP,CARD9,MAP2K1,NFKBIA,BIRC3,CXCL1,IL12B,RIPK1,TRAF6,CCL5,IL1B,CD14,IKBKB,LCN2,CXCL8,PRTN3,TICAM2,JUN,MAPK14,AKT1,IL6,IRAK3,LY96,IRF7,PIK3CA,TNF,MAP2K3,MAPK8,TLR6,HSP90AA1,MAPK3,IRAK1,MAP3K7,CXCL2,IRF5,PYCARD,RELA,MAP2K4,IFNA1/IFNA13,NLRP3,MAPK1,IFNB1,NLRC4 |
| Cellular Movement | 3.67E-34-2.38E-10 | JUN,MAPK14,TLR5,IL12A,AKT1,TNFRSF1A,TICAM1,TIRAP,NOD1,DMBT1,CARD9,MAP2K1,IL6,NFKBIA,IRAK3,LY96,IRF7,PIK3CA,TNF,CXCL1,IL12B,MAP2K3,MAPK8,HSP90AA1,MAPK3,TRAF6,MAP3K7,IRAK1,IL1B,CCL5,CD14,IKBKB,CXCL2,LCN2,IRF5,CXCL8,RELA,PYCARD,LYZ,PRTN3,MAP2K4,NLRP3,MAPK1,IFNB1,NLRC4 |
| Cellular Development | 4.7E-34-2.65E-10 | TLR5,IL12A,TNFRSF1A,TLR1,NOD1,TICAM1,TIRAP,CARD9,MAP2K1,NFKBIA,BIRC3,CXCL1,IL12B,RIPK1,TRAF6,CCL5,IL1B,CD14,IKBKB,LCN2,CXCL8,PRTN3,TICAM2,SUGT1,JUN,MAPK14,AKT1,IL6,IRAK3,LY96,IRF7,PIK3CA,TNF,MAP2K3,MAPK8,TLR6,HSP90AA1,MAPK3,IRAK1,MAP3K7,CXCL2,IRF5,RELA,PYCARD,MAP2K4,IFNA1/IFNA13,NLRP3,MAPK1,IFNB1,NLRC4 |
| ***C. jejuni/* 2 h post- infection** | | |
| Cell Death and Survival | 2.23E-51-5.93E-12 | IL12A,TNFRSF1A,TLR1,NOD1,TIRAP,CARD9,FADD,MAP2K1,NFKBIA,BIRC3,TLR4,CXCL1,XIAP,RIPK2,SLC11A1,TRAF6,CCL5,IL1B,CASP1,IKBKB,LCN2,CXCL8,LYZ,IL18,LTF,TICAM2,JUN,MAPK14,MYD88,AKT1,LBP,CAMP,IRAK3,IRF7,PIK3CA,TNF,MAPK8,TLR6,HSP90AA1,MAPK3,IRAK1,MAP3K7,NFKB1,CHUK,BPI,SLPI,CXCL2,IRF5,RELA,PYCARD,NLRP3,MAP2K4,IFNA1/IFNA13,MAPK1,IFNB1,RAC1,CASP8,NLRC4 |
| Cell-To-Cell Signaling and Interaction | 1.99E-50-6.26E-12 | IL12A,TLR1,TNFRSF1A,TIRAP,NOD1,CARD9,MAP2K1,FADD,NFKBIA,BIRC3,TLR4,CXCL1,RIPK2,SLC11A1,TRAF6,IL1B,CCL5,CASP1,IKBKB,PSTPIP1,LCN2,CXCL8,LYZ,IL18,LTF,ZBP1,JUN,MAPK14,MYD88,AKT1,LBP,CAMP,IRAK3,LY96,IRF7,PIK3CA,TNF,MAPK8,TLR6,HSP90AA1,MAPK3,IRAK1,MAP3K7,NFKB1,CHUK,BPI,SLPI,CXCL2,IRF5,PYCARD,RELA,NLRP3,IFNA1/IFNA13,MAP2K4,MAPK1,IFNB1,RAC1,CASP8 |
| Cellular Development | 4.92E-43-3.96E-12 | IL12A,TNFRSF1A,TLR1,TIRAP,NOD1,CARD9,FADD,MAP2K1,NFKBIA,BIRC3,TLR4,CXCL1,RIPK2,XIAP,TRAF6,CCL5,IL1B,CASP1,IKBKB,PSTPIP1,LCN2,CXCL8,IL18,LTF,TICAM2,SUGT1,JUN,MAPK14,AKT1,MYD88,CAMP,IRAK3,LY96,IRF7,PIK3CA,TNF,MAPK8,TLR6,HSP90AA1,MAPK3,IRAK1,MAP3K7,NFKB1,CHUK,BPI,SLPI,CXCL2,IRF5,RELA,PYCARD,MAP2K4,IFNA1/IFNA13,NLRP3,MAPK1,IFNB1,RAC1,CASP8,NLRC4 |
| Cellular Growth and Proliferation | 4.92E-43-6.26E-12 | IL12A,TNFRSF1A,TLR1,TIRAP,CARD9,FADD,MAP2K1,NFKBIA,BIRC3,TLR4,CXCL1,RIPK2,XIAP,TRAF6,CCL5,IL1B,CASP1,IKBKB,PSTPIP1,LCN2,CXCL8,LYZ,IL18,LTF,TICAM2,SUGT1,JUN,MAPK14,AKT1,MYD88,CAMP,IRAK3,LY96,IRF7,PIK3CA,TNF,MAPK8,TLR6,HSP90AA1,MAPK3,IRAK1,MAP3K7,NFKB1,CHUK,BPI,SLPI,CXCL2,IRF5,RELA,PYCARD,MAP2K4,IFNA1/IFNA13,MAPK1,IFNB1,RAC1,CASP8,NLRC4 |
| Cellular Function and Maintenance | 2.62E-39-6.93E-12 | IL12A,TLR1,TNFRSF1A,NOD1,TIRAP,CARD9,FADD,MAP2K1,NFKBIA,BIRC3,TLR4,CXCL1,RIPK2,XIAP,SLC11A1,TRAF6,CCL5,IL1B,CASP1,IKBKB,PSTPIP1,LCN2,CXCL8,IL18,LTF,TICAM2,JUN,MAPK14,MYD88,AKT1,CAMP,IRAK3,LY96,IRF7,PIK3CA,TNF,MAPK8,TLR6,HSP90AA1,MAPK3,IRAK1,MAP3K7,NFKB1,CHUK,BPI,SLPI,CXCL2,IRF5,PYCARD,RELA,MAP2K4,IFNA1/IFNA13,NLRP3,MAPK1,IFNB1,RAC1,NLRC4,CASP8 |
| ***C. jejuni/* 24 h post- infection** | | |
| Cell Death and Survival | 5.33E-48-5.38E-10 | IL12A,TNFRSF1A,TLR1,TICAM1,NOD1,TIRAP,CARD9,MAP2K1,NFKBIA,BIRC3,TLR4,CXCL1,XIAP,IL12B,RIPK2,SLC11A1,RIPK1,CCL5,IL1B,CD14,NOD2,CASP1,IKBKB,LCN2,CXCL8,LTF,TICAM2,JUN,MAPK14,AKT1,LBP,CAMP,IRF7,PIK3CA,TNF,TOLLIP,MAP2K3,HSP90AA1,IRAK1,NFKB1,CHUK,SLPI,CXCL2,IRF5,RELA,NLRP3,IFNA1/IFNA13,NLRC4,CASP8 |
| Cell-To-Cell Signaling and Interaction | 1.03E-41-3.64E-10 | TLR5,IL12A,TLR1,TNFRSF1A,TIRAP,TICAM1,NOD1,CARD9,MAP2K1,NFKBIA,BIRC3,TLR4,CXCL1,IL12B,RIPK2,SLC11A1,RIPK1,IL1B,CCL5,NOD2,CD14,CASP1,IKBKB,PSTPIP1,LCN2,CXCL8,LTF,JUN,MAPK14,AKT1,LBP,CAMP,LY96,IRF7,PIK3CA,TNF,TOLLIP,MAP2K3,HSP90AA1,IRAK1,NFKB1,CHUK,SLPI,CXCL2,IRF5,RELA,IFNA1/IFNA13,NLRP3,CASP8 |
| Cellular Movement | 1.2E-39-4.58E-10 | JUN,MAPK14,TLR5,IL12A,AKT1,TNFRSF1A,TICAM1,TIRAP,NOD1,LBP,CARD9,CAMP,MAP2K1,NFKBIA,TLR4,LY96,IRF7,PIK3CA,TNF,CXCL1,IL12B,RIPK2,MAP2K3,XIAP,SLC11A1,HSP90AA1,IRAK1,NFKB1,CHUK,IL1B,CCL5,NOD2,CD14,SLPI,CASP1,IKBKB,CXCL2,LCN2,IRF5,CXCL8,RELA,LTF,NLRP3,NLRC4,CASP8 |
| Cellular Function and Maintenance | 6.12E-39-4.47E-10 | TLR5,IL12A,TLR1,TNFRSF1A,TICAM1,NOD1,TIRAP,CARD9,MAP2K1,NFKBIA,BIRC3,TLR4,CXCL1,IL12B,RIPK2,XIAP,SLC11A1,RIPK1,IL1B,CCL5,NOD2,CD14,CASP1,IKBKB,PSTPIP1,LCN2,CXCL8,LTF,TICAM2,JUN,MAPK14,AKT1,CAMP,LY96,IRF7,PIK3CA,TNF,MAP2K3,HSP90AA1,IRAK1,NFKB1,CHUK,SLPI,CXCL2,IRF5,RELA,IFNA1/IFNA13,NLRP3,NLRC4,CASP8 |
| Cell Signaling | 1.03E-31-4.58E-10 | JUN,TLR5,MAPK14,AKT1,TNFRSF1A,TICAM1,TIRAP,NOD1,CARD9,CAMP,MAP2K1,NFKBIA,BIRC3,TLR4,PIK3CA,TNF,CARD6,XIAP,RIPK2,IL12B,MAP2K3,SLC11A1,HSP90AA1,RIPK1,IRAK1,CHUK,IL1B,CCL5,CD14,NOD2,CASP1,IKBKB,RELA,LTF,TICAM2,CASP8 |
| ***EcN+ C. jejuni/* 2 h post- infection** | | |
| Cell-To-Cell Signaling and Interaction | 1.19E-46-6.05E-11 | TLR5,IL12A,TLR1,TNFRSF1A,NOD1,TIRAP,CARD9,MAP2K1,FADD,NFKBIA,BIRC3,TLR4,CXCL1,SLC11A1,RIPK1,TRAF6,IL1B,CCL5,NOD2,CD14,CASP1,PSTPIP1,CXCL8,IL18,PRTN3,ZBP1,JUN,MAPK14,MYD88,AKT1,CAMP,IRAK3,IRF7,TNF,TOLLIP,MAP2K3,MAPK8,TLR6,HSP90AA1,MAPK3,NFKB1,CHUK,BPI,MPO,CXCL2,IRF5,PYCARD,RELA,IFNA1/IFNA13,MAPK1,IFNB1,RAC1 |
| Cell Death and Survival | 7.41E-45-5.89E-11 | IL12A,TNFRSF1A,TLR1,NOD1,TIRAP,CARD9,FADD,MAP2K1,NFKBIA,BIRC3,TLR4,CXCL1,XIAP,SLC11A1,RIPK1,TRAF6,IL1B,CCL5,CD14,NOD2,CASP1,CXCL8,IL18,PRTN3,TICAM2,JUN,MAPK14,AKT1,MYD88,CAMP,IRAK3,IRF7,TNF,TOLLIP,MAP2K3,MAPK8,TLR6,HSP90AA1,MAPK3,NFKB1,CHUK,BPI,MPO,CXCL2,IRF5,PYCARD,RELA,NAIP,IFNA1/IFNA13,MAPK1,IFNB1,RAC1 |
| Cellular Development | 6.98E-38-5.89E-11 | TLR5,IL12A,TNFRSF1A,TLR1,NOD1,TIRAP,CARD9,FADD,MAP2K1,NFKBIA,BIRC3,TLR4,CXCL1,XIAP,RIPK1,TRAF6,CCL5,IL1B,CD14,CASP1,PSTPIP1,CXCL8,IL18,PRTN3,TICAM2,SUGT1,JUN,MAPK14,AKT1,MYD88,CAMP,IRAK3,IRF7,TNF,MAP2K3,MAPK8,TLR6,HSP90AA1,MAPK3,NFKB1,CHUK,BPI,CXCL2,IRF5,RELA,PYCARD,IFNA1/IFNA13,MAPK1,IFNB1,RAC1 |
| Cellular Growth and Proliferation | 6.98E-38-5.89E-11 | TLR5,IL12A,TNFRSF1A,TLR1,TIRAP,CARD9,FADD,MAP2K1,NFKBIA,BIRC3,TLR4,CXCL1,XIAP,RIPK1,TRAF6,CCL5,IL1B,CD14,NOD2,CASP1,PSTPIP1,CXCL8,IL18,PRTN3,TICAM2,SUGT1,JUN,MAPK14,AKT1,MYD88,CAMP,IRAK3,IRF7,TNF,MAP2K3,MAPK8,TLR6,HSP90AA1,MAPK3,NFKB1,CHUK,BPI,MPO,CXCL2,IRF5,RELA,PYCARD,IFNA1/IFNA13,MAPK1,IFNB1,RAC1 |
| Cellular Function and Maintenance | 4.61E-36-3.77E-11 | TLR5,IL12A,TLR1,TNFRSF1A,NOD1,TIRAP,CARD9,MAP2K1,FADD,NFKBIA,BIRC3,TLR4,CXCL1,XIAP,SLC11A1,RIPK1,TRAF6,CCL5,IL1B,CD14,NOD2,CASP1,PSTPIP1,CXCL8,IL18,PRTN3,TICAM2,JUN,MAPK14,MYD88,AKT1,CAMP,IRAK3,IRF7,TNF,MAP2K3,MAPK8,TLR6,HSP90AA1,MAPK3,NFKB1,CHUK,BPI,MPO,CXCL2,IRF5,PYCARD,RELA,IFNA1/IFNA13,MAPK1,IFNB1,RAC1 |
| ***EcN+ C. jejuni/* 24 h post- infection** | | |
| Cell Death and Survival | 1.26E-58-6.41E-13 | IL12A,TNFRSF1A,TLR1,TICAM1,NOD1,TIRAP,CARD9,FADD,MAP2K1,NFKBIA,BIRC3,TLR4,CXCL1,XIAP,IL12B,RIPK2,SLC11A1,RIPK1,TRAF6,CCL5,IL1B,CD14,NOD2,CASP1,IKBKB,LCN2,CXCL8,LYZ,LTF,PRTN3,TICAM2,JUN,MAPK14,MYD88,AKT1,LBP,IL6,CAMP,IRAK3,IRF7,PIK3CA,TNF,TOLLIP,MAP2K3,MAPK8,TLR6,HSP90AA1,MAPK3,IRAK1,NFKB1,CHUK,MPO,SLPI,CXCL2,IRF5,RELA,NAIP,MAP2K4,IFNA1/IFNA13,MAPK1,NLRP1,IFNB1,RAC1,CASP8,NLRC4 |
| Cell-To-Cell Signaling and Interaction | 4.5E-55-4.85E-13 | TLR5,IL12A,TLR1,TNFRSF1A,TIRAP,TICAM1,NOD1,CARD9,MAP2K1,FADD,NFKBIA,BIRC3,TLR4,CXCL1,IL12B,RIPK2,SLC11A1,RIPK1,TRAF6,IL1B,CCL5,NOD2,CD14,CASP1,IKBKB,PSTPIP1,LCN2,CXCL8,LYZ,LTF,PRTN3,ZBP1,JUN,MAPK14,MYD88,AKT1,LBP,IL6,CAMP,IRAK3,LY96,IRF7,PIK3CA,TNF,TOLLIP,MAP2K3,MAPK8,TLR6,HSP90AA1,MAPK3,IRAK1,NFKB1,CHUK,MPO,SLPI,CXCL2,IRF5,RELA,MAP2K4,IFNA1/IFNA13,MAPK1,IFNB1,RAC1,CASP8 |
| Cellular Function and Maintenance | 3.53E-46-4.85E-13 | TLR5,IL12A,TLR1,TNFRSF1A,TICAM1,NOD1,TIRAP,CARD9,FADD,MAP2K1,NFKBIA,BIRC3,TLR4,CXCL1,IL12B,RIPK2,XIAP,SLC11A1,RIPK1,TRAF6,CCL5,IL1B,CD14,NOD2,CASP1,IKBKB,PSTPIP1,LCN2,CXCL8,LTF,PRTN3,TICAM2,JUN,MAPK14,MYD88,AKT1,IL6,CAMP,IRAK3,LY96,IRF7,PIK3CA,TNF,MAP2K3,MAPK8,TLR6,HSP90AA1,MAPK3,IRAK1,NFKB1,CHUK,MPO,SLPI,CXCL2,IRF5,RELA,MAP2K4,IFNA1/IFNA13,MAPK1,NLRP1,IFNB1,RAC1,NLRC4,CASP8 |
| Cellular Development | 5.44E-44-4.17E-13 | TLR5,IL12A,TNFRSF1A,TLR1,NOD1,TICAM1,TIRAP,CARD9,FADD,MAP2K1,NFKBIA,BIRC3,TLR4,CXCL1,RIPK2,IL12B,XIAP,RIPK1,TRAF6,CCL5,IL1B,CD14,CASP1,IKBKB,PSTPIP1,LCN2,CXCL8,LTF,PRTN3,TICAM2,SUGT1,JUN,MAPK14,AKT1,MYD88,IL6,CAMP,IRAK3,LY96,IRF7,PIK3CA,TNF,MAP2K3,MAPK8,TLR6,HSP90AA1,MAPK3,IRAK1,NFKB1,CHUK,SLPI,CXCL2,IRF5,RELA,MAP2K4,IFNA1/IFNA13,MAPK1,IFNB1,RAC1,CASP8,NLRC4 |
| Cellular Growth and Proliferation | 5.44E-44-4.17E-13 | TLR5,IL12A,TNFRSF1A,TLR1,TICAM1,TIRAP,CARD9,FADD,MAP2K1,NFKBIA,BIRC3,TLR4,CXCL1,RIPK2,IL12B,XIAP,RIPK1,TRAF6,CCL5,IL1B,CD14,NOD2,CASP1,IKBKB,PSTPIP1,LCN2,CXCL8,LYZ,LTF,PRTN3,TICAM2,SUGT1,JUN,MAPK14,AKT1,MYD88,IL6,CAMP,IRAK3,LY96,IRF7,PIK3CA,TNF,MAP2K3,MAPK8,TLR6,HSP90AA1,MAPK3,IRAK1,NFKB1,CHUK,MPO,SLPI,CXCL2,IRF5,RELA,MAP2K4,IFNA1/IFNA13,MAPK1,IFNB1,RAC1,CASP8,NLRC4 |

The overlap p‐value measures whether there is a statistically significant overlap between the dataset genes and the genes that are regulated by a TR.  An overlap p-value is computed based on significant overlap between genes in the dataset and known targets regulated by the transcriptional regulator. Overlap was calculated using Fisher’s Exact Test, and significance was assessed at p‐values < 0.01.
