# Supplementary figures and images for: Immuno-modulatory effect of probiotic E. coli Nissle 1917 in polarized human colonic cells against Campylobacter jejuni infection
Source: Gut Microbes. 2020 Dec 31;13(1):1857514. doi: 10.1080/19490976.2020.1857514 (PMC7781529; doi:10.1080/19490976.2020.1857514)

## Slide 1
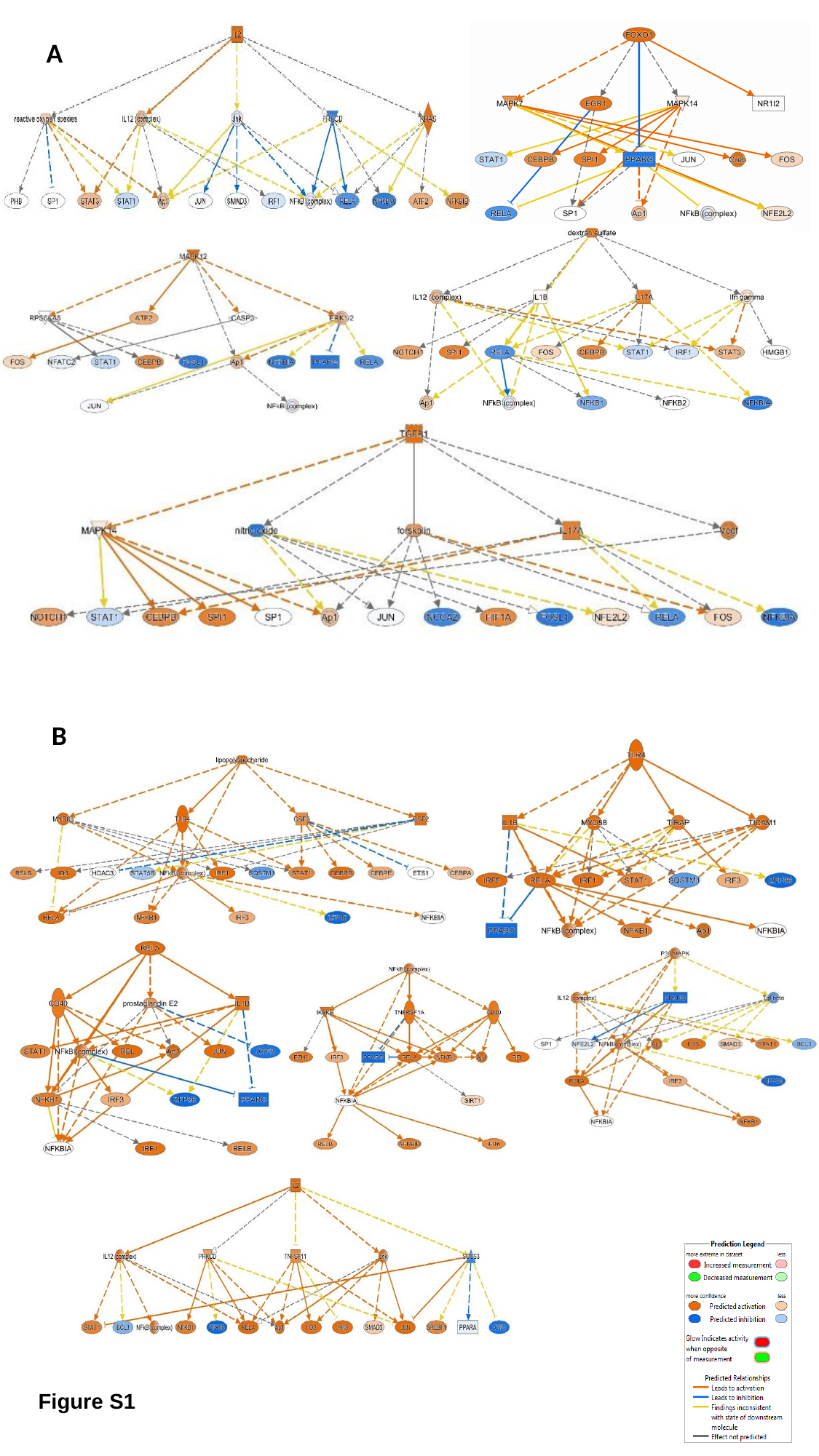

A
B
Figure S1

## Slide 2
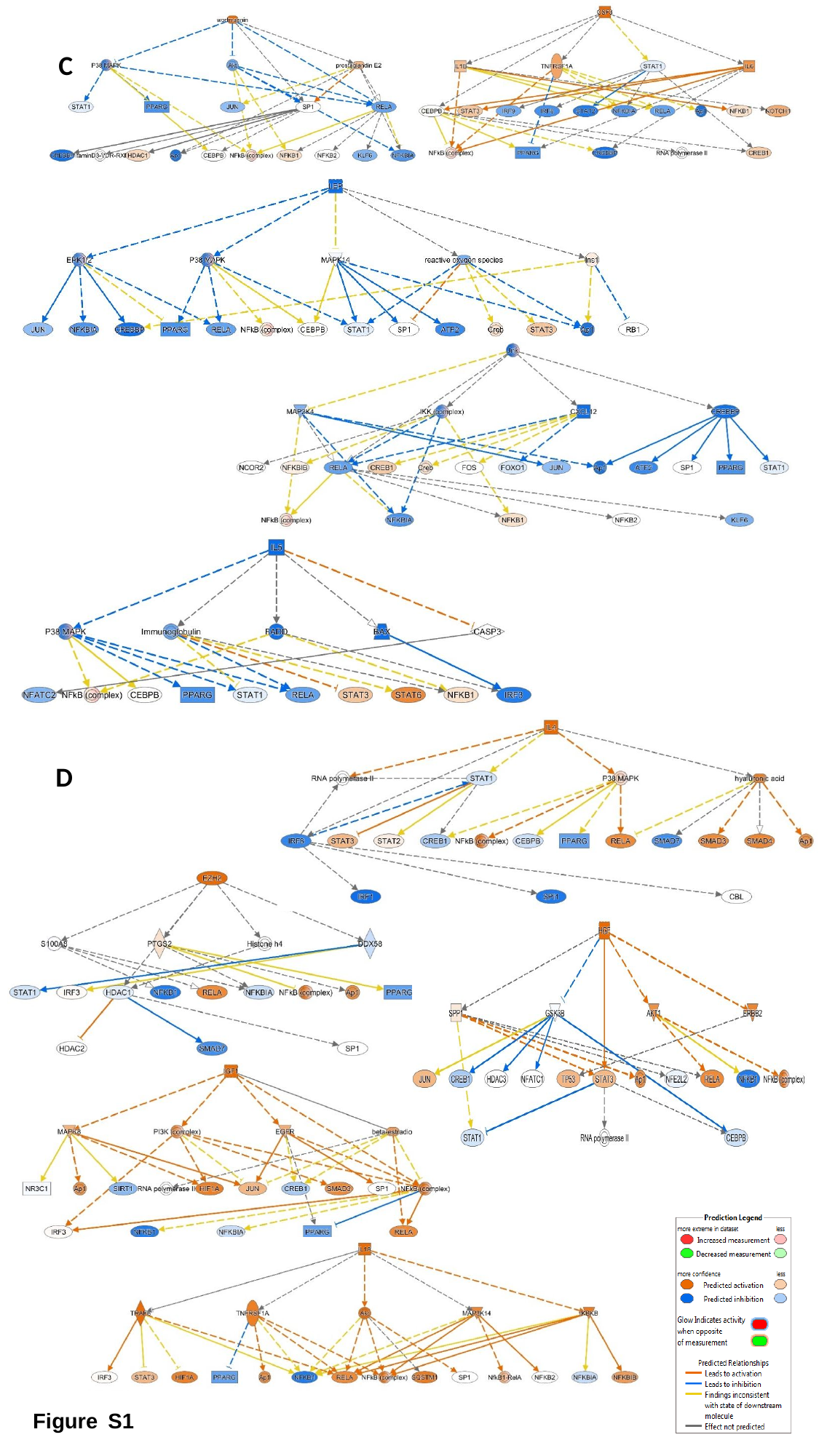

C
D
Figure S1

## Slide 3
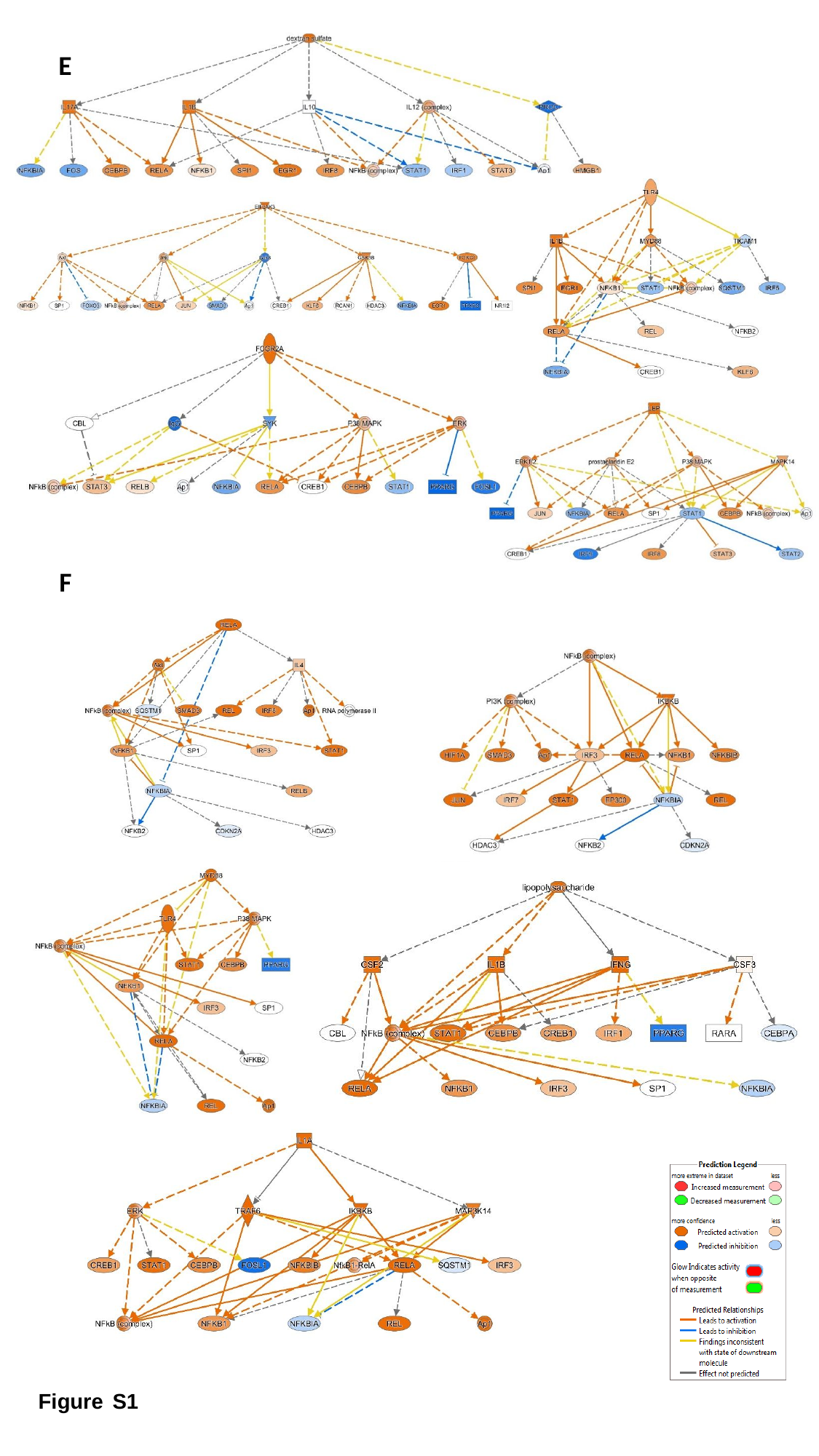

E
F
Figure S1

Supplement: Supplemental Material [file KGMI_A_1857514_SM8366.zip › Supplementary information/Supplementary figures.pptx]
